# Predicted and observed impacts of COVID-19 lockdowns: two Health Impact Assessments in Scotland and Wales

**DOI:** 10.1093/heapro/daac134

**Published:** 2022-11-11

**Authors:** Liz Green, Kathryn Ashton, Mark Bellis, Timo Clements, Margaret Douglas

**Affiliations:** Policy and International Health, WHO Collaborating Centre on ‘Investment in Health and Well-being’, Public Health Wales, Number 2 Capital Quarter, Tyndall Street, Cardiff CF10 4BZ, UK; Department of International Health, Care and Public Health Research Institute – CAPHRI, Maastricht University, Maastricht, The Netherlands; Policy and International Health, WHO Collaborating Centre on ‘Investment in Health and Well-being’, Public Health Wales, Number 2 Capital Quarter, Tyndall Street, Cardiff CF10 4BZ, UK; Department of International Health, Care and Public Health Research Institute – CAPHRI, Maastricht University, Maastricht, The Netherlands; Policy and International Health, WHO Collaborating Centre on ‘Investment in Health and Well-being’, Public Health Wales, Number 2 Capital Quarter, Tyndall Street, Cardiff CF10 4BZ, UK; Department of Public Health and Life Sciences, Bangor University, College Road, Bangor LL57 2DG, UK; Department of Public Health and Life Sciences, Bangor University, College Road, Bangor LL57 2DG, UK; Usher Institute, University of Edinburgh, Medical School, Teviot Place, Edinburgh EH8 9AG, UK; Public Health Scotland, Gyle Square, Edinburgh EH12 9EB, UK

**Keywords:** Health Impact Assessments, COVID-19, evaluation, social determinants of health

## Abstract

Health Impact Assessment is a key approach used internationally to identify positive or negative impacts of policies, plans and proposals on health and well-being. In 2020, HIAs were undertaken in Scotland and Wales to identify the potential health and well-being impacts of the ‘stay at home’ and physical distancing measures implemented at the start of the coronavirus disease (COVID-19) pandemic. There is sparse evidence evaluating whether the impacts predicted in HIAs occur following policy implementation. This paper evaluates the impacts anticipated in the COVID-19 HIAs against actual observed trends. The processes undertaken were compared and predicted impacts were tabulated by population groups and main determinants of health. Routine data and literature evidence were collated to compare predicted and observed impacts. Nearly all health impacts anticipated in both HIAs have occurred in the direction predicted. There have been significant adverse impacts through multiple direct and indirect pathways including loss of income, social isolation, disruption to education and services, and psychosocial effects. This research demonstrates the value of prediction in impact assessment and fills a gap in the literature by comparing the predicted impacts identified within the HIAs with observed trends. Post-COVID-19 recovery should centre health and well-being within future policies and decisions. Processes like HIA can support this as part of a ‘health in all policies’ approach to improve the health and well-being of populations.

## INTRODUCTION

Health Impact Assessment (HIA) is a key approach to promote ‘Health in all policies’ by assessing determinants of health and health equity likely to be affected by proposed policies, plans and proposals in all sectors. This should enable governments to ensure their policies promote health and health equity (Rogerson *et al.*, 2020). HIA aims to identify potential impacts—positive or negative, intended and unintended—their scale and nature, and affected populations. It uses evidence and follows the five steps (Green *et al.*, 2020; WHO, 2022) depicted in Box 1. HIA can support collaboration with stakeholders who provide insights and ensure ownership of action. (Leppo *et al.*, 2013; WHO, 2014; Rogerson *et al.*, 2020; Green *et al.*, 2021).

**Box 1: The HIA process**

Step 1: Screening to determine whether to complete an HIA. This includes consideration of whether there are likely to be effects on health and whether there is scope for changes to improve these impacts.

Step 2: Scoping the boundaries of the assessment, including timeframes, resources, key stakeholders to engage with, evidence collection methods and key determinants and populations of focus.

Step 3: Appraisal of evidence, which is triangulated and analysed. This evidence can include peer-reviewed and grey literature, stakeholder evidence and routinely gathered statistics and data for example, government statistics and reporting.

Step 4: Recommendations and reporting to inform decision makers, including the construction of a report which includes the findings and any recommended actions that should be taken to maximize the positive impact and mitigate any negative impact.

Step 5: Review and reflection including monitoring and evaluation. This involves highlighting milestones to measure any changes in impact or if the predicted impacts were observed, reviewing the process and any impact which it may have had on decisions and future policies.

HIAs and other impact assessments are best used to inform policies before decisions are taken and involve anticipating or predicting their potential impacts (Haigh *et al.*, 2013). While prospective assessment is needed to inform decision-making (Davenport *et al.*, 2006), the lack of direct evidence can lead some to question the validity of findings (Parry and Stevens, 2001). Retrospective validation of prediction in impact assessment is little addressed in the literature and discussion has focussed on the use of predictive tools (Hecky *et al.*, 1984; George, 2000; Gontier *et al.*, 2006; Molefe, 2017) or the challenges of evaluating and predicting impacts (Ali *et al.*, 2009). For prediction in HIA specifically, the literature is even sparser (Petticrew *et al.*, 2007; Veerman *et al.*, 2007).

Evaluations of HIA can consider their accuracy in predicting the health and well-being impacts; how well stakeholders were engaged or their effectiveness in informing decisions (Parry and Kemm, 2005). Several studies have evaluated the effectiveness of HIAs in influencing decisions (Wismar *et al.* 2007; Dannenberg *et al.*, 2008; Haigh *et al.*, 2013, 2015; Nour *et al.*, 2016; Bias and Abildso, 2017; Buregeya *et al.*, 2020). Some studies have evaluated the HIA process including the evidence used (Tyler *et al.*, 2019), consideration of equity (Povall *et al.*, 2014; Buregeya *et al.*, 2019) or level of participation (Thondoo *et al.*, 2019). But we found none that assessed the accuracy of prediction by comparing the impacts predicted in an HIA with those observed after a policy or plan has been implemented.

The coronavirus disease (COVID-19) pandemic and the measures taken to control transmission, including requirements to stay at home, work from home, and socially distance, have had far-reaching effects on populations and society since March 2020 (Gautam and Hens, 2020; Public Health England, 2020; WHO, 2020; World Economic Forum, 2020; Chiesa *et al.*, 2021; United Nations, 2021). These have affected health and inequalities beyond the direct morbidity and mortality caused by illness from the virus (Dyakova *et al.*, 2021). They have affected wider determinants of health, including the economy, the environment, social interaction, mental well-being and access to services such as education and health (Dahlgren and Whitehead, 2021). They have also had differential effects on population groups including older people, children, young people, women and those on low incomes (UK Government, 2020; United Nations, 2020).

In 2020, during the first wave of COVID-19 in the UK, both the Scottish Health and Inequalities Impact Assessment Network (SHIIAN) and the Wales Health Impact Assessment Support Unit (WHIASU) carried out HIAs to identify the potential impacts of ‘stay at home’ and physical distancing measures (‘lockdown’) that were then being implemented (Douglas *et al.*, 2020; Green *et al.*, 2021a). Table 1 presents the policy measures that were assessed.

**Table 1:** Lockdown policy measures in Wales and Scotland assessed in the HIAs

Wales	Scotland
The Welsh HIA was completed rapidly from 1 April to 11 May 2020, as the regulations were coming into force. These included the following: •Welsh Ministers, registered public health officials and police constables had the right to detain people infected or contaminated with coronavirus •required some business premises to close, and required those that were allowed to stay open, such as supermarkets, to put specific measures in place to ensure adequate social distancing •restricted the movements of individuals so that they were not allowed to leave the place they were living without a ‘reasonable excuse’. The regulations included examples of these such as shopping for food, exercising once a day, getting medical help and travelling to work where it was not reasonable and practicable to work from home •closed places of worship, apart from in limited circumstances such as in relation to funerals •required Natural Resources Wales, local authorities, National Park Authorities and the National Trust to close public footpaths and access land, where the use of a path or land posed a high risk of spreading coronavirus.	The Scottish HIA was completed rapidly between 13 and 20 March 2020, before restrictions were approved or implemented. The authors assumed that measures to contain the virus would include all of some of the following: •Advising the whole population to self-isolate at home if they or their family are symptomatic •Bans on social gatherings •Stopping flights and public transport •Closure of ‘non-essential’ workplaces (i.e. beyond the health and social care sector, utilities and the food chain) with continued working from home for those that can •Closure of schools, colleges and universities •Prohibition of all ‘non-essential’ population movement •Limiting contact for special populations (i.e. care homes, prisons)

Both HIAs considered potential impacts on population health, well-being and health inequalities and made recommendations to mitigate negative impacts and enhance positive impacts. This paper aims to compare the predicted impacts identified in the two HIAs, with observed trends in both countries over the 18-month period after the start of the pandemic, between March 2020 and December 2021. The paper briefly compares the two HIAs, reports on a comparison of predicted and observed impacts, their accuracy, discusses reasons for any differences, and suggests implications for policy and practice.

## METHODS

The methods and findings of both the Welsh and the Scottish HIAs have been reported previously (Douglas *et al.*, 2020, 2020a; Green *et al.*, 2020a, 2021a). Both assessed the ‘stay at home’ and physical or social distancing policies of the Welsh and Scottish Governments, and implemented in response to the COVID-19 pandemic to control the transmission of the virus. They followed the standard HIA process (Douglas, 2011; Bhatia *et al.*, 2014; Winkler *et al.*, 2020; WHO, 2022) and were undertaken in the first wave of the pandemic (March–May 2020).

### Overview of the Scottish and Welsh HIAs

Table 2 summarizes the processes followed in the two HIAs. The Scottish HIA was completed in 1 week between 13 and 20 March 2020, before lockdown measures were initiated on 26 March. The HIA used a health impact checklist (Douglas, 2019) to identify potential mechanisms through which positive and negative impacts might arise. The five authors then collated routine data, systematic reviews, and other peer-reviewed and grey literature. They estimated for each impact an approximate number of people likely to be affected, and a qualitative description of severity and direction of the impacts (positive or negative). There was no stakeholder engagement. The report presented the main areas of impact and recommended mitigation measures (Douglas *et al.*, 2020a) and included a more detailed impacts table. The HIA was used as an initial framework and subsequently developed further as part of the Public Health Scotland COVID-19 Response.

**Table 2:** The Welsh and Scottish HIA processes

	Scottish HIA	Welsh HIA
Stages of the HIA	1.Screening 2.Scoping 3.Appraisal of evidence 4.Recommendations 5.Reporting	1.Screening 2.Scoping 3.Appraisal of evidence 4.Recommendations 5.Reporting 6.Process review and reflection
Timing of the HIA	Immediately prior to the introduction of COVID-19 regulations	As regulations were introduced
Checklists used	•Population groups •Wider determinants of health	•Health and well-being determinants •Population groups
Characterization of each impact	•Affected populations •Estimated number affected •Severity	•Positive/negative •Severity/duration/likelihood
Evidence used to inform the HIA	•Routine data •Public health knowledge •Background information •Literature review—mainly systematic reviews	•Health intelligence and data •Public health knowledge •Grey literature and background information •Literature review—systematic reviews and other peer-reviewed articles •Stakeholder involvement
Stakeholder evidence	None	15 interviews were undertaken with key stakeholders

The Welsh HIA was completed in 2 months between April and 11 May 2020, from the start of lockdown and during the first few weeks of the first wave. The HIA used health and well-being and population group checklists (WHIASU, 2020) to identify mechanisms through which impacts might arise. The six authors collated routine data, systematic reviews, and peer-reviewed and grey literature. Stakeholder engagement involved interviews with 15 key stakeholders from Wales. The HIA team estimated for each impact the direction (positive/negative), likelihood, intensity/severity and duration using previously validated definitions (Green *et al.*, 2020). Reporting included an Executive Summary; a Main Report with an analysis of main areas of impact, recommended mitigation measures, and detailed impacts table; and a Supporting Information technical report (Green *et al.*, 2020a). The HIA was used as an initial framework with an indication of the scale of some impacts and subsequently developed further as part of the Public Health Wales and Welsh Government’s COVID-19 Response. This paper uses the areas of impact highlighted in the original HIA report.

Overall, the two HIAs followed similar processes and assessed broadly similar policies. However, the Welsh HIA gathered evidence from stakeholders and included a more comprehensive literature review and evidence-gathering process. The Scottish HIA was carried out immediately before the lockdown, whilst the Welsh HIA was conducted during the first 8 weeks. It also included a review and reflection on the process and recommended monitoring of future impacts.

### Comparing HIA predictions with observed impacts

Firstly, the affected populations and main determinants or areas of impact identified in the two HIA reports were tabulated to compare the impacts identified between each HIA. Robust routine monitoring and survey data on relevant trends were then collated for each identified area of impact. This included Scottish and Welsh Government data, data from StatsWales, the Office for National Statistics, Public Health Wales reports and surveys such as the PHW Public Engagement Survey on Coronavirus Measures (Public Health Wales, 2022), Public Health Scotland reports and surveys. The authors also drew on other surveys in Wales Scotland-and UK-wide. These sources were used to compare the direction of change for each determinant with the direction (positive or negative) predicted in the HIAs. We did not attempt to assess whether the judgments of severity were accurate as these were mostly qualitative descriptors. We graded the strength of evidence available for each impact as High (rated as 1) for government or public health institute data and for peer-reviewed papers and Low for other sources (rated as 2).

## RESULTS

### Comparison of predicted and observed impacts

Tables 3 and 4 present a summary of the predicted impacts and whether they were observed and accurate, indicating the strength of supporting evidence. Supplementary Tables S1 and S2 provide further detail. For most of the impacts, high-grade evidence was available from government or public health institute sources supporting the prediction. For both HIAs, most predicted impacts were observed in the expected direction (positive or negative). As predicted, there were negative impacts on income, particularly for people already on low incomes, which were only partially mitigated by the Coronavirus Job Retention scheme and other measures (Widnall *et al.*, 2020; ONS, 2021). Social isolation led to high levels of loneliness (Groarke *et al.*, 2020; Public Health Wales, 2020; WCPP, 2021; Scottish Government, 2022) and there is evidence of increased child abuse and domestic abuse (Davenport *et al.*, 2020; ONS, 2020; Scottish Government, 2020, 2021b), as predicted in both HIAs. The HIAs predicted that disruption and unwillingness to attend health settings would affect the care of non-COVID-19 conditions, and this has occurred (Scottish Public Health Observatory, 2021; Welsh Parliament, 2021a). Similarly, the HIAs predicted disruption to education would increase educational inequalities and there is evidence that this has happened (Public Health Wales, 2021; Scottish Government, 2021). The predicted short-term fall in car and other journeys occurred, resulting in improved air quality and other environmental impacts (Transport Scotland, 2021; Welsh Parliament, 2021b). But, as also predicted, reluctance to use public transportation has led to higher car travel and this now accounts for a higher proportion of journeys. Both HIAs predicted negative effects on mental well-being through high levels of anxiety (Public Health Wales, 2020; Scottish Government, 2020a; Mind Cymru, 2021). They also predicted positive effects on community cohesion through collective individual and neighbourhood responses and there is evidence of high levels of community support during the pandemic (Edinburgh Community Health Forum, 2020; Public Health Wales, 2020). The HIAs predicted potential negative impacts on ethnic minority populations and there has been an observed increase in hate crime and reports of harassment affecting people of Asian ancestry and disabled people (BBC, 2021; UK Government, 2021). The Welsh HIA predicted exacerbation of these impacts by crowded living conditions, and there is evidence of an increase in household disputes (Woodfine *et al.*, 2021). The Scottish HIA identified the negative impact of restricted access to greenspace, and surveys found that use of greenspace reduced during the 2020 lockdown period although respondents reported it benefited their mental health (Public Health Scotland, 2021a).

**Table 3:** Predicted and observed health impacts of the COVID-19 lockdowns on population groups

Population group	Predicted health impacts identified in Scottish HIA	Predicted health impacts identified in Welsh HIA	Prediction confirmed as observed? (strength of evidence^a^)	
			Scotland	Wales
Older people	Negative impact—high-risk of infection and social isolation	Negative impact—social isolation	✓ (1)	✓ (1)
Children and young people	Negative impact—disrupted education	Negative impact—disrupted education, adverse childhood experiences, reduced socializations, loss of employment for young people due to shutdown sectors	✓ (1)	✓ (1)
Women	Negative impact—more likely to be carers, income loss if need to provide childcare during school closures, potential for increase in family violence	Negative impact—more likely to be carers, potential for increase in family violence, stress from patient and public facing roles, loss of employment from shutdown sectors	✓ (1)	✓ (1)
Minority ethnic groups	Negative impact—increased discrimination and harassment	Negative impact—worse health outcomes from infection, increased discrimination and harassment	✓ (2)	✓ (1)
People with existing mental health issues	Negative impact—social isolation, risk of relapse/withdrawal in substance misusers	Negative impact—worsening mental well-being	✓ (1)	✓ (1)
Those with disabilities including learning challenges	Negative impact—disrupted support services	Negative impact—disrupted support services. Positive impact—more opportunities to join the workforce	✓ (1)	✓ (1)
Homeless people	Negative impact—disrupted support service, unable to self-isolate	Positive impact—providing shelters to prevent transmission could end street sleeping	✓ (1)	✓ (1)
People in criminal justice system	Negative impact—difficulty of isolation in prison setting, loss of contact with family	No predicted impacts identified	✓ (1)	–
Undocumented migrants	Negative impact—may have no access or be reluctant to engage with health services	No predicted impacts identified	No data available	–
Workers	Negative impact—workers on precarious contracts or self-employed may be at significant risk of adverse impacts from loss of work and no income	Negative impact—those who work in sectors which have closed due to restrictions, resulting in people losing jobs or experiencing reduced income, increased mental health impacts on key workers	✓ (1)	✓ (1)
People with low income	Negative impact— already more likely to have poor health	Negative impact—financial insecurity, although mitigated by the furlough scheme	✓ (1)	✓ (1)
People in institutions	Negative impact—care homes, special needs facilities, prisons, migrant detention centres, cruise liners—these institutions may act as amplifiers	Negative impact—care homes may act as amplifiers	✓ (1)	✓ (1)

^a^Strength of evidence: (1) High—evidence of trends from national statistics, government or public health institute reports or peer-reviewed papers. (2) Low—no national statistics or peer-reviewed papers but evidence available from other surveys, grey literature reports or other sources.

**Table 4:** Predicted and observed health impacts of the COVID-19 lockdowns on the wider determinants of health

Determinant of health and well-being/ Pathway of impact	Predicted health impacts in Scottish HIA	Predicted health impacts in Welsh HIA	Prediction confirmed as observed? (Strength of evidence^a^)	
			Scotland	Wales
Economic impacts	Negative impact—loss of income, increased unemployment and recession	Negative impact— loss of income and spending, increased unemployment, and closure of small businesses Positive impact—home working has provided greater flexibility	✓ (1)	✓ (1)
Social isolation	Negative impact—individuals who live alone and have lower access to digital connectivity	Negative impact—all populations groups affected Positive impact—digital technology lead to increased connectivity	✓ (1)	✓ (1)
Food security	Negative impact—difficulty accessing food and other supplies	Positive impact—protection of the vulnerable for example through free school meals	✓ (1)	✓ (1)
Family relationships	Negative impact—home confinement may increase family violence and abuse	Negative impact—potential for domestic abuse	✓ (1)	✓ (1)
Health-related behaviours	Negative impact—substance misuse, gambling, unintended pregnancies, reduced physical activity	Negative impact—substance misuse, snacking Positive impact—increased appreciation of the importance of physical activity	✓ (1)	✓ (1) ✓ (1)
Disruption to essential services	Negative impact—health and social care demand, unwillingness to attend healthcare setting, loss of workforce	Negative impact—reduced access to services	✓ (1)	✓ (1)
Disruption to education	Negative impact—loss of education and skills, reliance on home schooling	Negative impact—loss of education and skills	✓ (1)	✓ (1)
Traffic, transport and green and blue infrastructure	Positive impact—reduced traffic and air pollution in short-term Negative impact—restricted public transport, reduced use of public transport, restricted access to green space	Positive impact—reduced traffic and air pollution in short-term Negative impact—reduced use of public transport	✓ (1)	✓ (1)
Social disorder	Negative impact—potential for unrest	Positive impact—reduction in crime rates, increased trust in police	✓ (1)	✓ (1)
	Negative impact—harassment of people believed to be at risk of transmitting the virus		✓ (1)	
Psychosocial impacts	Negative impact—public fear and anxiety	Negative impact—public fear and anxiety	✓ (1)	✓ (1)
	Positive impact—community cohesion	Positive impact—mobilization of society	✓ (1)	✓ (1)
Living conditions		Negative impact—Crowded or poor housing quality exacerbating existing health conditions Positive impact -There has been rapid action to place those who are homeless in accommodation.	-	✓ (1)

^a^Strength of evidence: (1) High—Evidence of trends from national statistics, government or public health institute reports or peer-reviewed papers. (2) Low—No national statistics or peer-reviewed papers but evidence available from other surveys, grey literature reports or other sources

The Scottish HIA identified the potential for unrest, whereas the Welsh HIA predicted a reduction in overall crime and increased trust in the police. The Welsh HIA was more correct, as most forms of crime reduced and there has been no significant unrest in either nation, although there were increases in some types of crime such as domestic abuse, as noted above, and virtual fraud (Public Health Wales, 2020; Scottish Government, 2020, 2021a). The HIAs also differed in their assessment of likely impacts on health-related behaviour, and the observed impacts have also been mixed ([Bibr CIT0057][Bibr CIT0057]; Public Health Wales, 2020). Both HIAs identified the potential for increases in health-harming behaviours, for example snacking and alcohol use. The Scottish HIA predicted a reduction in physical activity whereas the Welsh HIA predicted that increased appreciation of physical activity may lead some to increase physical activity. The available data suggest that some people adopted healthier behaviours, but a higher proportion adopted less healthy behaviours, including physical activity for which larger declines were seen in younger, ethnic minority and unemployed populations (Obesity Action Scotland, 2020; Public Health Wales, 2020). The Welsh HIA also identified a reduction in street sleeping (Woodfine *et al.*, 2021) and increased digital connectivity (Public Health Wales, 2020), both of which occurred, partly due to mitigation measures implemented early in the pandemic.

Both HIAs identified population groups that would be particularly vulnerable to negative impacts. Most of these populations have been disproportionately affected by the anticipated impacts. The only exception is that both HIAs predicted that older people would bear a higher impact on mental health than other groups. Despite bearing a disproportionately much higher burden of direct morbidity and mortality from COVID-19, older people have reported lower levels of anxiety, loneliness and hopelessness than younger age groups (Office for Health Improvement and Disparities, 2021).

## DISCUSSION

This paper has shown that most of the health impacts anticipated in the Scottish and Welsh HIAs have occurred in the predicted direction, using monitoring data up to December 2021. As predicted, there have been significant adverse impacts through multiple pathways including loss of income and employment; mental health and well-being impacts of social isolation, stress and anxiety; family stress and increased violence against women and domestic abuse; disruption to health and other services; educational disruption; and a reluctance to use public transport. These have disproportionately affected population groups who were already disadvantaged, for example, women, older people, those on low incomes, children and young people. The impacts on health-related behaviours have been more mixed, for example some people increased physical activity or alcohol intake, and some reduced it. The main positive impact identified in the Scottish HIA was the potential for an increased sense of community, which has been demonstrated. The Welsh HIA identified further positive impacts reflecting mitigation measures early in the pandemic, such as provision of accommodation for homeless people and the increased use of digital technology.

We are not aware of any other evaluations of HIAs that assessed whether the anticipated impacts proved to be correct after the proposed policy was implemented. Assessing the predictive accuracy of an HIA is difficult, as the impacts that occur after implementation may be altered by responses taken after the HIA has been completed (Parry and Stevens, 2001; Petticrew *et al.*, 2007). This includes measures taken to implement the HIA recommendations, so ironically if an HIA is effective in influencing policy this makes it more difficult to evaluate the accuracy of its predictions. In this case, several interventions were implemented to help mitigate wider impacts, although not directly as a result of these HIAs. These include the Coronavirus Job Retention Scheme (‘furlough’) providing economic support to employers and employees (House of Commons Library, 2021a, 2021b), a temporary increase in universal credit (Department for Work and Pensions, 2021) and providing free laptops to some children and young people to support online learning (Department for Education, 2020). These measures will have mitigated some of the anticipated negative impacts, but significant residual impacts still clearly occurred, for example those on low incomes bore strongly negative impacts despite employment support (Mental Health Foundation, 2020).

Both HIAs followed the standard HIA process but differed in timing and depth. The earlier timing of the Scottish HIA potentially gave more opportunity to inform early mitigation measures, whereas the later Welsh HIA allowed early evidence of emerging impacts to suggest changes to mitigate or maximize these. The Scottish HIA was completed very rapidly in 1 week and included relevant data and literature evidence but no stakeholder involvement. The more comprehensive Welsh HIA allowed a fuller characterization of the likelihood, significance and duration of each impact, using criteria validated in previous HIAs (Green *et al.*, 2020).

Finally, the HIAs identified very similar impacts with some differences (Tables 3 and 4; Supplementary Tables S1 and S2). The Welsh HIA identified more opportunities for positive health and well-being. This included the potential for home working to promote better work/life balance and flexible working; increased family connectivity; and the potential to develop a more sustainable economic model prioritizing health and well-being. The main area in which they reached opposing conclusions was the impact on crime. The Welsh HIA predicted a crime reduction, based on police reports during the early part of the pandemic. Conversely, the Scottish HIA predicted a potential increase in crime and social disorder due to discontent about the pandemic response and reduced policing capacity. In practice, there was little significant disorder in the UK (although events elsewhere show the potential for this to arise). Crime overall fell during restrictions, especially violent crimes associated with the night-time economy, but some crimes such as domestic abuse, child abuse and virtual fraud increased (Scottish Government, 2020; Office for National Statistics, 2022). A more detailed HIA in Scotland may have predicted these nuances. Both HIAs identified the potential for an increase in health-harming behaviours, but they diverged in their prediction of impacts on physical activity. Whereas the Scottish HIA identified the potential for reduced physical activity because of closed sports facilities and less utilitarian active travel, the Welsh HIA identified increased appreciation of the importance of physical activity as population movements outside of the home were restricted (PHW, 2020). In both countries and across the UK, the impacts on health-related behaviours have been mixed, with polarization between populations and a likely increase in inequalities (Convention of Scottish Local Authorities and Scottish Government, 2020; Public Health England, 2020a). Some negative consequences were not foreseen by either HIA, i.e. the Welsh HIA identified positive opportunities for local tourism but not the accompanying negative impacts of increased tourist traffic, increased accommodation prices and environmental damage (Schofields Insurance, 2020; Christian, 2021; Department for Transport, 2021).

The strengths of this evaluation are that it is original in evaluating the accuracy of predictions in the HIAs, and the broad range of data sources used to assess whether anticipated impacts emerged in the direction expected. There are limitations to the available data for some impacts, particularly health-related behaviours and differences between population groups. Also, some impacts may emerge later—for example, longer-term impacts on unemployment, predicted by both HIAs, are not yet clear. The HIAs presented a high-level characterization of different types of impacts and the evaluation has not sought to determine whether judgments about scale or severity of impact were correct. The authors were involved in the HIAs, which might bias our conclusions. However, the two HIAs were completed independently and this evaluation, involving authors of both HIAs, encouraged cross-scrutiny and appraisal and used robust sources where available. As noted above, anticipated impacts will have been affected by mitigation measures and further work is needed to explore the extent to which the HIAs were able to influence policy responses.

### Implications for HIA practice

This evaluation demonstrates that even a very rapid HIA can correctly predict many relevant impacts on health and equity before a policy is implemented. The breadth of the impacts and their differential effects reinforce the need for a holistic approach, enabling HIAs to identify potential impacts affecting different populations through multiple pathways. Comparison between the HIAs demonstrates the benefit of stakeholder involvement and a more detailed review of supporting evidence. These are routinely advocated to enable robust analysis (Mindell *et al.*, 2004, 2010, 2014; Tamburrini *et al.*, 2011; Negev *et al.*, 2013). Evidence from stakeholders, who were being affected by impacts that had not yet been captured in literature or statistics, provided more certainty and depth. In every HIA, there is a need to balance the available resources and capacity, the level of detail needed to provide robust conclusions and the need for timeliness of recommendations to influence decision-making and inform actions.

Both HIAs were used as a framework for initial mitigation and were then developed further. This allowed the initial findings to be used while emerging impacts were explored. This shows that HIAs can support an ongoing, collaborative ‘Health in All Policies’ approach working across sectors rather than being just a one-off assessment (Rogerson *et al.*, 2020; Green *et al.*, 2021).

This evaluation shows the potential to monitor observed impacts following an HIA, using routine data. Although most HIA guidance suggests that HIA should include monitoring and evaluation (Quigley and Taylor, 2004; Harris *et al.*, 2007; Dannenberg *et al.*, 2008; National Research Council (US) Committee on Health Impact Assessment, 2011; Pyper *et al.*, 2021), in practice this is often not done. Undoubtedly, many of the impacts anticipated in these HIAs were partially mitigated by other measures implemented alongside social distancing restrictions. However, this paper shows that it is still possible to assess whether the HIA predictions were broadly correct. Further similar evaluations could help demonstrate whether, and when, HIAs are effective in predicting future impacts and improve future practice. This paper assessed national-level HIAs for which national data were readily available, but relevant data are also often disaggregated to more local levels thus making this approach replicable locally. HIAs should highlight the priority indicators to monitor, so they can be collated following proposal implementation.

The close match between predicted and observed negative impacts raises the question of whether the HIAs were effective in informing action to mitigate these. Were the HIAs ignored, or did they inform actions that in practice only partially reduced the impacts? The legal context is important as the ability to more fully mitigate some impacts may have been beyond the powers of the devolved Welsh and Scottish governments. A consideration for future HIAs is to prioritize the impacts that are most amenable to action and specify the authorities with power to implement these. Further research could explore how HIAs are used by policy-makers to enhance their effectiveness in influencing action.

In both HIAs, some or all authors worked in the national public health organization. This enabled access to relevant evidence, for example, health observatory data and statistics, but also more importantly allowed the findings to be used in the relevant organizations’ responses to the wider impacts of the pandemic. Both HIAs and findings were also published in academic journals, reaching a wider audience (Douglas *et al.*, 2020; Green *et al.*, 2021a).

### Implications for policy

HIAs are themselves an intervention that aim to influence the outcome of the policies assessed (Mindell *et al.*, 2014). The finding that even very rapid HIAs, such as the Scottish example, can effectively predict a wide range of impacts on health also supports the more widespread use of HIA in policymaking. Routine use of HIA could identify unanticipated potential health impacts before, or as, policies are implemented, offering the opportunity to mitigate adversely and enhance positive impacts in ‘real time’ (Green *et al.*, 2021a). Evaluations showing evidence of validated prediction should increase commissioners’ confidence to use HIA.

The data also highlight the range of negative impacts of the pandemic and their differential effects. There is a clear need for continuing action to address these residual health impacts in the post-pandemic period. Processes such as HIA can help to ensure actions are well designed to enhance their positive effects, avoid unanticipated harms and are targeted to the populations most affected.

## CONCLUSION

This paper evaluated the impacts identified in two HIAs that assessed the impact of ‘lockdown’ in Scotland and Wales in 2020. It demonstrates the value of prediction in HIA and fills a gap in the literature by comparing predicted with observed impacts. The rapid Scottish and more comprehensive Welsh approaches both have value, with the stakeholder involvement and more comprehensive evidence review allowing more detailed characterization of the impacts to inform decisions and action.

The pandemic has raised the profile of public health more widely. The use of processes such as HIA can build on this and inform decisions based on evidence and predictive analysis. Evaluations like this could increase confidence in prospective HIA. Post-COVID-19 recovery and renewal should allow health and well-being should be centred within future policies and decisions. Processes such as HIA can support this and form a key part of a ‘health in all policies’ approach (Wismar *et al.*, 2013).

## Supplementary Material

daac134_suppl_Supplementary_Material

## References

[CIT0001] Ali, S., O’Callaghan, V., Middleton, J. and Little, R. (2009) The challenges of evaluating a health impact assessment. Critical Public Health, 19, 171–180.

[CIT0002] BBC. (2021). COVID in Scotland: People Are Treating Us Like the Disease. https://www.bbc.co.uk/news/uk-scotland-edinburgh-east-fife-56113045 (16 March 2022, date last accessed).

[CIT0003] Bhatia, R., Farhang, L., Heller, J., Lee, M., Orenstein, M., Richardson, M. et al. (2014). Minimum Elements and Practice Standards for Health Impact Assessment, Version 3. https://hiasociety.org/resources/Documents/HIA-Practice-Standards-September-2014.pdf (9 March 2022, date last accessed).

[CIT0004] Bias, T. K. and Abildso, C. G. (2017) Measuring policy and related effects of a health impact assessment related to connectivity. Preventive Medicine, 95S, S92–S94. doi: 10.1016/j.ypmed.2016.08.007 27509869

[CIT0005] Buregeya, J. M., Loignon, C. and Brousselle, A. (2019) Contribution to healthy places: risks of equity free health impact assessment. Evaluation and Program Planning, 73, 138–145. doi: 10.1016/j.evalprogplan.2018.12.007 30622062

[CIT0006] Buregeya, J. M., Loignon, C. and Brousselle, A. (2020) Contribution analysis to analyze the effects of the health impact assessment at the local level: a case of urban revitalization. Evaluation and Program Planning, 79, 101746. doi: 10.1016/j.evalprogplan.2019.101746 31835151

[CIT0007] Chiesa, V., Antony, G., Wismar, M. and Rechel, B. (2021) COVID-19 pandemic: health impact of staying at home, social distancing and ‘lockdown’ measures-a systematic review of systematic reviews. Journal of Public Health (Oxford, England), 43, e462–e481. doi: 10.1093/pubmed/fdab102 PMC8083256 33855434

[CIT0008] Christian, A. (2021). Staycation Statistics and Trends for 2020, 2021 and 2022. https://www.snaptrip.com/c/information/staycation-statistics/ (10 March 2022, date last accessed).

[CIT0009] Convention of Scottish Local Authorities and Scottish Government. (2020). Scotland’s Wellbeing: the Impact of COVID-19. https://nationalperformance.gov.scot/sites/default/files/documents/NPF_Impact_of_COVID-19_December_2020.pdf (5 April 2022, date last accessed).

[CIT0010] Dahlgren, G. and Whitehead, M. (2021) The Dahlgren–Whitehead model of health determinants: 30 years on and still chasing rainbows. Public Health, 199, 20–24. 34534885 10.1016/j.puhe.2021.08.009

[CIT0011] Dannenberg, A. L., Bhatia, R., Cole, B. L., Heaton, S. K., Feldman, J. D. and Rutt, C. D. (2008) Use of health impact assessment in the U.S.: 27 case studies, 1999–2007. American Journal of Preventive Medicine, 34, 241–256. doi: 10.1016/j.amepre.2007.11.015. 18312813

[CIT0012] Davenport, C., Mathers, J. and Parry, J. (2006) Use of health impact assessment in incorporating health considerations in decision making. Journal of Epidemiology and Community Health, 60, 196–201. 16476747 10.1136/jech.2005.040105 PMC2465566

[CIT0013] Davenport, M. H., Meyer, S., Meah, V. L., Strynadka, M. C. and Khurana, R. (2020) Moms are not OK: COVID-19 and maternal mental health. Frontiers in Global Womens Health, 1, 1. doi: 10.3389/fgwh.2020.00001. PMC8593957 34816146

[CIT0014] Department for Education. (2020) Get Help with Technology for Remote Education. https://www.gov.uk/guidance/get-help-with-technology-for-remote-education (16 March 2022, date last accessed).

[CIT0015] Department for Transport. (2021) Provisional Road Traffic Estimates, Great Britain: October 2020 to September 2021. https://www.gov.uk/government/statistics/provisional-road-traffic-estimates-great-britain-october-2020-to-september-2021/provisional-road-traffic-estimates-great-britain-october-2020-to-september-2021 (5 April 2022, date last accessed).

[CIT0016] Department for Work and Pensions. (2021). £1000 Boost for Nearly 2m Working Households on Universal Credit. https://www.gov.uk/government/news/1000-boost-for-nearly-2m-working-households-on-universal-credit (16 March 2022, date last accessed).

[CIT0017] Douglas, M. (2011) Health impact assessments: principles and practice. Journal of Public Health4, 33, 365. https://doi-org.mu.idm.oclc.org/10.1093/pubmed/fdr073.

[CIT0018] Douglas, M. (2019) Health Impact Assessment Guidance for Practitioners. https://www.scotphn.net/wp-content/uploads/2015/11/Health-Impact-Assessment-Guidance-for-Practitioners-SHIIAN-updated-2019.pdf (10 March 2022, date last accessed)

[CIT0019] Douglas, M., Katikireddi, S. V., Taulbut, M., McKee, M. and McCartney, G. (2020) Mitigating the wider health effects of COVID-19 pandemic response. BMJ (Clinical Research Ed.), 369, m1557. doi: 10.1136/bmj.m1557. 10.1136/bmj.m1557 PMC7184317 32341002

[CIT0020] Douglas, M., Katikireddi, S. V., Taulbut, M., McKee, M. and McCartney, G. (2020a). Health Impacts of Physical Distancing Measures in Scotland. Rapid Health Impact Assessment. https://www.scotphn.net/wp-content/uploads/2015/11/HIA_social_distancing-LONG-VERSION-final.pdf (10 March 2022, date last accessed).

[CIT0021] Dyakova, M., Couzens, L., Allen, J., Van Eimeren, M., Stielke, A., Cotter-Roberts, A. et al. (2021) Placing Health Equity at the Heart of the COVID-19 Sustainable Response and Recovery: Building Prosperous Lives for All in Wales. The Welsh Health Equity Status Report Initiative (WHESRi). https://phw.nhs.wales/news/placing-health-equity-at-the-heartof-coronavirus-recovery-for-building-a-sustainable-future-for-wales/placing-healthequity-atthe-heart-of-the-covid-19-sustainable-response-and-recovery-building-prosperous-lives-forall-in-wales/ (28 May 2021, date last accessed).

[CIT0022] Edinburgh Community Health Forum. (2020) The Contribution of Edinburgh Community Health Forum Member Organisations to the COVID-19 Response. http://www.echf.org.uk/wp-content/uploads/2020/11/The-contribution-of-ECHF-to-the-COVID-19-response.pdf (16 March 2022, date last accessed).

[CIT0023] Gautam, S. and Hens, L. (2020) COVID-19: impact by and on the environment, health and economy’. Environment, Development and Sustainability, 22, 4953–4954 https://doi-org.mu.idm.oclc.org/10.1007/s10668-020-00818-7 32837275 10.1007/s10668-020-00818-7 PMC7324289

[CIT0024] George, C. (2000) Chapter 5: Environmental impact prediction and evaluation. In Lee, N. and George, C. (eds), Environmental Assessment in Developing and Transitional Countries: Principles, Methods and Practice. John Wiley and Sons, Ltd, Chichester.

[CIT0025] Gontier, M., Balfors B. and Mortberg, U. (2006) Biodiversity in environmental assessment – current practice and tools for prediction. Environmental Impact Assessment Review, 26, 268–286.

[CIT0026] Green, L., Ashton, K., Azam, S., Dyakova, M., Clements, T. and Bellis, M. (2021a) Using health impact assessment (HIA) to understand the wider health and well-being implications of policy decisions: the COVID-19 ‘staying at home and social distancing policy’ in Wales. BMC Public Health, 21, 1456. doi: 10.1186/s12889-021-11480-7 34315469 PMC8313659

[CIT0027] Green, L., Ashton, K., Bellis, M. A., Clemens, T. and Douglas, M. (2021) Health in All Policies – a key driver for health and well-being in a post-COVID-19 pandemic world’. International Journal of Environmental Research and Public Health, 18, 9468. doi: 10.3390/ijerph18189468 34574390 PMC8468680

[CIT0028] Green, L., Ashton, K., Edmonds, N. and Azam, S. (2020) Process, practice and progress: a case study of the Health Impact Assessment (HIA) of Brexit in Wales. International Journal of Environmental Research and Public Health, 17, 6652. doi: 10.3390/ijerph17186652 PMC7557572 32932632

[CIT0029] Green, L., Morgan, L., Azam, S., Evans, L., Parry-Williams, L., Petchey, L. et al. (2020a) A Health Impact Assessment of the ‘Staying at Home and Social Distancing Policy’ in Wales in Response to the COVID-19 Pandemic. https://phw.nhs.wales/news/staying-at-home-policy-has-reduced-spread-of-coronavirus-but-has-also-had-other-positive-and-negative-impacts-on-the-well-being-of-welsh-society/a-health-impact-assessment-of-the-staying-at-home-and-social-distancing-policy-in-wales-in-response-to-th/ (9 March 2022, date last accessed).

[CIT0030] Groarke, J. M., Berry, E., Graham-Wisener, L., McKenna-Plumley, P. E., McGlinchey, E. and Armour, C. (2020) Loneliness in the UK during the COVID-19 pandemic: cross-sectional results from the COVID-19 psychological wellbeing study. PLoS One, 15, e0239698. doi: 10.1371/journal.pone.0239698 32970764 PMC7513993

[CIT0031] Haigh, F., Baum, F., Dannenberg, A. L., Harris, M. F., Harris-Roxas, B. and Keleher, H. (2013) The effectiveness of health impact assessment in influencing decision-making in Australia and New Zealand 2005–2009. BMC Public Health, 13, 1188. doi: 10.1186/1471-2458-13-1188 24341545 PMC3878483

[CIT0032] Haigh, F., Harris, E., Harris-Roxas, B. et al. (2015) What makes health impact assessments successful? Factors contributing to effectiveness in Australia and New Zealand. BMC Public Health, 15, 1009. doi: 10.1186/s12889-015-2319-8 26433492 PMC4592749

[CIT0033] Harris, P., Harris-Roxas, B., Harris, E. and Kemp, L. (2007). Health Impact Assessment: A Practical Guide, Sydney: Centre for Health Equity Training, Research and Evaluation (CHETRE). Part of the UNSW Research Centre for Primary Health Care and Equity, UNSW. https://hiaconnect.edu.au/wp-content/uploads/2012/05/Health_Impact_Assessment_A_Practical_Guide.pdf (9 March 2022, date last accessed).

[CIT0034] Hecky, R. E., Newbury, R. W., Bodaly, R. A., Patalas, K. and Rosenberg, D. M. (1984) Environmental impact prediction and assessment: the Southern Indian lake experience. Canadian Journal of Fisheries and Aquatic Sciences, 41, 4.

[CIT0035] House of Commons Library. (2021a) Coronavirus Job Retention Scheme: Statistics. https://commonslibrary.parliament.uk/research-briefings/cbp-9152/ (16 March 2022, date last accessed).

[CIT0036] House of Commons Library. (2021b) Food Banks in the UK. https://researchbriefings.files.parliament.uk/documents/CBP-8585/CBP-8585.pdf (16 March 2022, date last accessed).

[CIT0037] Leppo, L., Ollila, E., Pena, S., Wismar, M. and Cook, S. (eds) (2013). Health in All Policies. Seizing Opportunities, Implementing Policies. https://www.euro.who.int/__data/assets/pdf_file/0007/188809/Health-in-All-Policies-final.pdf (9 March 2022, date last accessed).

[CIT0038] Mental Health Foundation. (2020). Coronavirus: The Divergence of Mental Health Experiences During the Pandemic. https://www.mentalhealth.org.uk/sites/default/files/Coronavirus%20Scotland%20-%20The%20divergence%20of%20mental%20health%20experiences%20%28Final%29_0.pdf (16 March 2022, date last accessed).

[CIT0039] Mind Cymru. (2021). Coronavirus: The Consequences for Mental Health in Wales. https://www.mind.org.uk/media/8961/the-consequences-of-coronavirus-for-mental-health-in-wales-final-report.pdf (24 March 2022, date last accessed).

[CIT0040] Mindell, J., Biddulph, J., Taylor, L., Lock, K., Boaz, A., Joffe, M. et al. (2010) Improving the use of evidence in health impact assessment. Bulletin of the World Health Organization, 88, 543–550. doi: 10.2471/BLT.09.068510 20616974 PMC2897984

[CIT0041] Mindell, J., Boaz, A., Joffe, M., Curtis, S. and Birley, M. (2004) Enhancing the evidence base for health impact assessment. Journal of Epidemiology & Community Health, 58, 546–551. 15194713 10.1136/jech.2003.012401 PMC1732829

[CIT0042] Mindell, J., Sheridan, L., Joffe, M., Samson-Barry, H. and Atkinson, S. (2014) Health impact assessment as an agent of policy change: improving the health impacts of the mayor of London’s draft transport strategy. Journal of Epidemiology & Community Health, 58, 169–174. 10.1136/jech.2003.012385 PMC1732710 14966225

[CIT0043] Molefe, N.M. (2017). Effective Impact Prediction: How Accurate Are Predicted Impacts in EIAs? https://wiredspace.wits.ac.za/handle/10539/23570#:~:text=Of%20all%20the%20impacts%20predicted%20in%20the%20reports%2C,soil%20pollution%2C%20fires%20and%20loss%20of%20agricultural%20potential (5 April 2022, date last accessed).

[CIT0044] National Research Council (US) Committee on Health Impact Assessment. (2011) Improving Health in the United States: The Role of Health Impact Assessment. National Academies Press, Washington, DC. 22379655

[CIT0045] Negev, M., Davidovitch, N., Garb, Y. and Tal, A. (2013) Stakeholder participation in health impact assessment: a multicultural approach. Environmental Impact Assessment Review, 43, 112–120.

[CIT0046] Nour, K., Dutilly-Simard, S., Brousselle, A., Smits, P., Buregeya, J. M., Loslier, J. et al. (2016) Evaluation of the effects of health impact assessment practice at the local level in Monteregie. Health Research Policy System, 14, 7, doi: 10.1186/s12961-016-0076-5. PMC4729162 26818241

[CIT0047] Obesity Action Scotland. (2020) Lifestyle of Scotland’s People since the Coronavirus Outbreak: Summary Report. https://www.obesityactionscotland.org/media/1467/polling-summary-report-2805.pdf (16 March 2022, date last accessed).

[CIT0048] Office for Health Improvement and Disparities. (2021) 3. Measures of Anxiety, Depression, Loneliness and Life Satisfaction. https://www.gov.uk/government/publications/covid-19-mental-health-and-wellbeing-surveillance-report/3-triangulation-comparison-across-surveys (16 March 2022, date last accessed). 10.1136/bmj.n2323 34561222

[CIT0049] Office for National Statistics. (2020) Domestic Abuse During the Coronavirus (COVID-19) Pandemic, England and Wales: November 2020. https://www.ons.gov.uk/peoplepopulationandcommunity/crimeandjustice/articles/domesticabuseduringthecoronaviruscovid19pandemicenglandandwales/november2020 (24 March 2022, date last accessed).

[CIT0050] Office for National Statistics. (2021) Labour Market Overview, UK: February 2021. https://www.ons.gov.uk/employmentandlabourmarket/peopleinwork/employmentandemployeetypes/bulletins/uklabourmarket/february2021#:~:text=In%20January%202021%2C%20726%2C000%20fewer,lower%20than%20the%20previous%20quarter (24 March 2022, date last accessed).

[CIT0051] Office for National Statistics. (2022) Crime in England and Wales: Year Ending September 2021. https://www.ons.gov.uk/peoplepopulationandcommunity/crimeandjustice/bulletins/crimeinenglandandwales/latest (10 March 2022, date last accessed).

[CIT0052] Parry, J. M. and Kemm, J. R. (2005) Evaluation of Health Impact Assessment workshop. Criteria for use in the evaluation of health impact assessments. Public Health, 119, 1122–1129, doi: 10.1016/j.puhe.2005.05.002 16329170

[CIT0053] Parry, J. and Stevens, A. (2001) Prospective health impact assessment: pitfalls, problems, and possible ways forward. BMJ, 323, 1177. doi: 10.1136/bmj.323.7322.117 11711414 PMC1121649

[CIT0054] Petticrew, M., Cummins, S., Sparks, L. and Findlay, A. (2007) Validating health impact assessment: prediction is difficult (especially about the future). Environmental Impact Assessment Review, 27, 101–107.

[CIT0055] Povall, S. L., Haigh, F. A., Abrahams, D. and Scott-Samuel, A. (2014) Health equity impact assessment. Health Promotion International, 29, 621–633, doi:10.1093/heapro/dat012. 23449601

[CIT0056] Public Health England. (2020) Wider Impacts of COVID-19 on Health. https://fingertips.phe.org.uk/profile/covid19 (8 March 2022, date last accessed).

[CIT0057] Public Health Scotland. (2021) Changes in Alcohol Consumption in Scotland During the Early Stages of the COVID-19 Pandemic: Descriptive Analysis of Repeat Cross-Sectional Survey Data. Available at: https://www.publichealthscotland.scot/media/2983/changes-in-alcohol-consumption-in-scotland-during-the-early-stages-of-the-covid-19-pandemic.pdf (16 March 2022, date last accessed).

[CIT0058] Public Health England. (2020a) Wider Impacts of COVID-19 on Physical Activity, Deconditioning and Falls in Older Adults. https://assets.publishing.service.gov.uk/government/uploads/system/uploads/attachment_data/file/1010501/HEMT_Wider_Impacts_Falls.pdf (5 April 2022, date last accessed).

[CIT0059] Public Health Scotland. (2021a) COVID-10 Green and Open Space Use in Spring 2021 (Wave 3). https://www.gla.ac.uk/media/Media_805950_smxx.pdf (16 March 2022, date last accessed).

[CIT0060] Public Health Wales. (2020) Public Engagement Survey on Health and Wellbeing during Coronavirus Measures. [Online]. https://phw.nhs.wales/topics/latest-information-on-novel-coronavirus-covid-19/how-are-you-doing/weekly-hayd-reports/week6-report-how-are-we-doing-in-wales/ (23 June 2020, date last accessed).

[CIT0061] Public Health Wales. (2021) Children and Young People’s Mental Well-Being During the COVID-19 Pandemic. https://phw.nhs.wales/publications/publications1/children-and-young-peoples-mental-well-being-during-the-covid-19-pandemic-report/ (24 March 2022, date last accessed).

[CIT0062] Public Health Wales. (2022) Public Health Wales Public Engagement Survey. https://phw.nhs.wales/news/public-health-wales-public-engagement-survey/ (9 March 2022, date last accessed).

[CIT0063] Pyper, R., Cave, B., Purdy, J. and McAvoy, H. (2021) Health Impact Assessment Guidance: A Manual. Standalone Health Impact Assessment and Health in Environmental Assessment. https://publichealth.ie/hia/guidance.pdf (9 March 2022, date last accessed).

[CIT0064] Quigley, R. J. and Taylor, L. C. (2004) Evaluating health impact assessment. Public Health, 118, 544–552. doi: 10.1016/j.puhe.2003.10.012 15530933

[CIT0065] Rogerson, B., Lindberg, R., Baum, F., Dora, C., Haigh, F., Simoncelli, A. M. et al. (2020) Recent advances in Health Impact Assessment and Health in All Policies implementation: lessons from an international convening in Barcelona. International Journal of Environmental Research and Public Health, 17, 7714. doi: 10.3390/ijerph17217714 PMC7659966 33105669

[CIT0066] Schofields Insurance. (2020) Report: The Rise of Staycations – UK Travel in 2020/21. https://www.schofields.ltd.uk/blog/5980/staycations-uk-travel-2020-21/ (5 April 2022, date last accessed).

[CIT0067] Scottish Government. (2020) Impact of COVID-19 on Crime. https://www.gov.scot/news/impact-of-covid-19-on-crime/ (10 March 2022, date last accessed).

[CIT0068] Scottish Government. (2020a) Coronavirus (COVID-19): Impact on Wellbeing – Research. https://www.gov.scot/publications/impact-covid-19-wellbeing-scotland/pages/5/ (16 March 2022, date last accessed).

[CIT0069] Scottish Government. (2021) Achievement of Curriculum for Excellence (CfE) Levels 2020-21. https://www.gov.scot/publications/achievement-curriculum-excellence-cfe-levels-2020-21/ (16 March 2022, date last accessed).

[CIT0070] Scottish Government. (2021a) Recorded Crime in Scotland, 2020-21. https://www.gov.scot/publications/recorded-crime-scotland-2020-2021/pages/3/ (16 March 2022, date last accessed).

[CIT0071] Scottish Government. (2021b) Domestic abuse: statistics recorded by the Police in Scotland – 2020/21. Available at: https://www.gov.scot/publications/domestic-abuse-recorded-police-scotland-2020-21/pages/4/ (16 March 2022, date last accessed).

[CIT0072] Scottish Government. (2022) Coronavirus in Scotland: Loneliness. https://data.gov.scot/coronavirus-covid-19/detail.html#loneliness (16 March 2022, date last accessed).

[CIT0073] Scottish Public Health Observatory. (2021) Covid-19 Wider Impacts. Available at: https://www.scotpho.org.uk/comparative-health/coronavirus-covid-19/covid-19-wider-impacts/ (25 October 2022, date last accessed).

[CIT0074] Tamburrini A. L. , Gilhuly, K. and Harris-Roxas, B. (2011) Enhancing benefits in health impact assessment through stakeholder consultation. Impact Assessment and Project Appraisal, 29, 195–204, doi:10.3152/146155111X12959673796281

[CIT0075] Thondoo, M., Rojas-Rueda, D., Gupta, J., de Vries, D. H. and Nieuwenhuijsen, M. J. (2019) Systematic literature review of health impact assessments in low and middle-income countries. International Journal of Environmental Research and Public Health, 16, 2018. doi:10.3390/ijerph16112018 PMC6603924 31174273

[CIT0076] Transport Scotland. (2021) COVID-19 Transport Trend Data – 20 August–5 September 2021. https://www.transport.gov.scot/publication/covid-19-transport-trend-data-30-august-5-september-2021/ (16 March 2022, date last accessed).

[CIT0077] Tyler, I., Pauly, B., Wang, J., Patterson, T., Bourgeault, I. and Manson, H. (2019) Evidence use in equity focused health impact assessment: a realist evaluation. BMC Public Health, 19, 230. doi: 10.1186/s12889-019-6534-6 30808317 PMC6390302

[CIT0078] UK Government. (2020) State of the Nation 2020: Children and Young People’s Well-being. https://assets.publishing.service.gov.uk/government/uploads/system/uploads/attachment_data/file/925329/State_of_the_nation_2020_children_and_young_people_s_wellbeing.pdf (9 March 2022, date last accessed).

[CIT0079] UK Government. (2021) Hate crime, England and Wales, 2020 to 2021. https://www.gov.uk/government/statistics/hate-crime-england-and-wales-2020-to-2021/hate-crime-england-and-wales-2020-to-2021 (24 March 2022, date last accessed).

[CIT0080] United Nations. (2020) Policy Brief: The Impact of COVID-19 on Women. https://www.un.org/sites/un2.un.org/files/policy_brief_on_covid_impact_on_women_9_april_2020.pdf (9 March 2022, date last accessed).

[CIT0081] United Nations. (2021) United Nations Comprehensive Response to COVID-19. https://www.un.org/sites/un2.un.org/files/un-comprehensive-response-covid-19-2021.pdf (8 March 2022, date last accessed).

[CIT0082] Veerman, J. L., Mackenbach, J. P. and Barendregt, J. J. (2007) Validity of predictions in health impact assessment. Journal of Epidemiology & Community Health, 61, 362–366. 17372299 10.1136/jech.2006.047480 PMC2652952

[CIT0083] Wales Centre for Public Policy. (2021) Loneliness in Wales during the Coronavirus Pandemic. https://www.wcpp.org.uk/wp-content/uploads/2021/10/Loneliness-in-Wales-during-the-Coronavirus-pandemic.pdf (24 March 2022, date last accessed).

[CIT0084] Welsh Parliament. (2021a). Impact of the Waiting Times Backlog on People in Wales Who Are Waiting for Diagnosis or Treatment. https://business.senedd.wales/mgIssueHistoryHome.aspx?IId=38257#:~:text=The%20waiting%20lists%20for%20diagnostic%20and%20therapy%20appointments,start%20treatment%20have%20been%20waiting%20over%209%20months (24 March 2022, date last accessed).

[CIT0085] Welsh Parliament. (2021b) Putting the ‘Public’ Back Into Public Transport. https://research.senedd.wales/research-articles/putting-the-public-back-into-public-transport/ (24 March 2022, date last accessed).

[CIT0086] WHIASU. (2020) Population Health Group Checklists. https://phwwhocc.co.uk/whiasu/wp-content/uploads/sites/3/2021/05/WHIASU_Population_Groups_Checklist.pdf (10 March 2022, date last accessed).

[CIT0087] Widnall, E., Winstone, L., Mars, B., Haworth, C., and Kidger, J. (2020). Young People’s Mental Health During the COVID-19 Pandemic. https://sphr.nihr.ac.uk/wp-content/uploads/2020/08/Young-Peoples-Mental-Health-during-the-COVID-19-Pandemic-Report.pdf (24 March 2022, date last accessed).

[CIT0088] Winkler, M. S., Furu, P., Viliani, F., Cave, B., Divall, M., Ramesh, G. et al. (2020) Current global health impact assessment practice. International Journal of Environmental Research and Public Health, 17, 2988. doi: 10.3390/ijerph17092988 PMC7246701 32344882

[CIT0089] Wismar, M., Blau, J., Ernst, K. and Figueras, J. (eds) (2007) The Effectiveness of Health Impact Assessment. WHO Regional Office for Europe, Copenhagen. https://apps.who.int/iris/bitstream/handle/10665/326506/9789289072960-eng.pdf?sequence=3&isAllowed=y (9 March 2022, date last accessed).

[CIT0090] Wismar, M., Kemm J. and Fehr, R. (2013) HIA supports ‘Health in all policies’. European Journal of Public Health, 23(Suppl 1).

[CIT0091] Woodfine, L., Green, L., Evans, L., Parry-Williams, L., Heathcote-Elliott, C., Grey, C. et al. (2021) No Place Like Home? Exploring the Health and Well-Being Impact of COVID-19 on Housing and Housing Insecurity. https://phw.nhs.wales/publications/publications1/no-place-like-home-summary-report/ (24 March 2022, date last accessed).

[CIT0092] World Economic Forum. (2020) The COVID-19 Lockdown Will Take Its Own Toll on Health, Researchers Warn. https://www.weforum.org/agenda/2020/04/how-the-covid-19-lockdown-will-take-its-own-toll-on-health/ (9 March 2022, date last accessed).

[CIT0093] World Health Organization. (2014) Health in Impact Assessments. Opportunities not to Be Missed. https://www.euro.who.int/__data/assets/pdf_file/0011/261929/Health-in-Impact-Assessments-final-version.pdf (9 March 2022, date last accessed).

[CIT0094] World Health Organization. (2020). Impact of COVID-19 on People’s Livelihoods, Their Health and Our Food Systems. https://www.who.int/news/item/13-10-2020-impact-of-covid-19-on-people’s-livelihoods-their-health-and-our-food-systems (9 March 2022, date last accessed).

[CIT0095] World Health Organization. (2022). Health Impact Assessment. https://www.who.int/heli/impacts/hiabrief/en/ (9 March 2022, date last accessed).

