# Supplementary material for: Predicted and observed impacts of COVID-19 lockdowns: two Health Impact Assessments in Scotland and Wales
Source: Health Promot Int. 2022 Nov 11;37(6):daac134. doi: 10.1093/heapro/daac134 (PMC9651036; doi:10.1093/heapro/daac134)
Supplement: daac134_suppl_Supplementary_Material [file daac134_suppl_supplementary_material.docx]

**Supplementary Table S1: Predicted and observed health impacts of the COVID-19 lockdowns on population groups**

| **Population Group** | **Predicted health impacts identified in Scottish HIA** | **Predicted health impacts identified in Welsh HIA** | **Observed health impacts in Scotland** | **Observed health impacts in Wales** |
| --- | --- | --- | --- | --- |
| Older people | - Highest direct risk of severe COVID-19 disease, more likely to live alone, less likely to use online communications, at risk of social isolation | - Mental well-being impact from social isolation, fear of the virus, disconnection from family and friends. Challenges obtaining food and essential supplies. | Death rate related to Covid-19 rises steeply with age National Records of Scotland, 2022).  But mental health impacts less in older than younger people – older age groups higher ‘happiness’ score with less anxiety, loneliness and hopelessness (Scottish Government, 2020; Mental Health Foundation, 2020). | Mortality and morbidity rates higher in older ages, and with co-morbidity of dementia. Older people more likely to be digitally excluded (Age Cymru, 2020; Wu, 2020; Hajek and König, 2021). |
| Children and young people | - Impact of disrupted education at critical time, longer term most at risk of poor employment and associated health outcomes in economic downturn | - The closure of childcare settings and schools has meant that some children may have lost access to a place of safety. Children could be at greater risk of adverse childhood experiences (ACEs) due to a range of factors dependent on the family situation. - Babies, children and young people (including young adults) have had their education interrupted, have experienced major changes to their routines and structures and have experienced reduced opportunities for socialising with peers. Children from low-income households are more likely to have been adversely affected. - Employment and income issues due to many YP working in sectors shut down such as non-essential retail | Deterioration in sleep, behaviour and eating of 2–7-year-old children reported by parents. 50% reported worse behaviour, 10% better; 50% worse mood, 10% better; 40% worse concentration, 10% better (Watson et al, 2020).  Young people have high levels of mental health symptoms (Scottish Government, 2020) – in April 2020 40% felt lonely (Mental Health Foundation, 2020), 39% were concerned about their own mental wellbeing and 61% about impact of coronavirus on their future (Youth Link Scotland, 2020). Less likely to say they could rely on neighbours for help (Scottish Government, 2020).  Declining overall primary school attainment, increasing attainment gap (Scottish Government, 2021) - figures below under disruption to education. | Increase in reported cases of grooming; increase in reported poorer mental wellbeing outcomes; increase in children exposed to ACEs (Cresswell et al, 2021; BMJ, 2021).  Young people affected by loss of employment and economic opportunities during lockdowns (ONS, 2021; Welsh Government, 2021).  Impact on mental wellbeing a major negative impact with small numbers reporting better mental health.  Children in low-income families impacted more (BBC, 2021a; Widnall et al, 2020). |
| Women | - More likely to be carers, likely to lose income if need to provide childcare during school closures, potential for increase in family violence for some | - Women and children who are more likely to have experienced violence including domestic violence and sexual abuse. - Women more likely to be carers - Women more likely to work in patient and public facing roles leading to increased stress and burnout - More likely to work in sectors closed down such as hospitality and non-essential retail | Women reported lower ‘happiness’ score and more anxiety, less likely to report worried about finances but more likely to be worried about lack of social contact and children’s education (Scottish Government, 2020).  Data on domestic abuse below. | Women experienced more negative impact on wellbeing although men had higher morbidity and mortality from COVID-19 (ONS, 2021a).  Women more likely to be care givers and responsible for home- schooling (Refuge, 2021; IFS, 2020).  Women more likely to be furloughed or lost job/ became unemployed and loss of income (ONS, 2021).  New mothers increase in depression and isolation  Increase in reports of violence against women, domestic abuse and sexual violence (Davenport et al, 2020; ONS, 2020). |
| Minority ethnic groups | - People of East Asian ethnicity – may be at risk of discrimination and harassment | - Black, Asian and Minority Ethnic (BAME) groups, who have been identified as having worse health outcomes as a result of contracting COVID-19, and who may also have experienced an increase in hate crimes. | Reported increase in hate crimes – see below.  Reports of harassment and abuse against people with SE Asian ethnicity (BBC, 2021).  Increased risk of death from Covid-19 in non-white ethnic groups (Public Health Scotland, n.d.). | Minority ethnic groups have poorer health outcomes and higher levels of morbidity and mortality compared to non-minority groups (Welsh Government, 2020).  Increase in hate crime reported during the pandemic (UK Government, 2021). |
| People with existing mental health issues | - People with mental health issues – may be at greater risk of impacts of social isolation - People who use substances or in recovery – risk of relapse or withdrawal | - Worsening of mental well-being for the whole population (such as depression and feelings of confusion, anger, anxiety and loneliness), as well as an exacerbation of mental health conditions. Those most at risk include women, women with children, those on low incomes, healthcare workers, those with existing mental health conditions, those who have been shielded and older people. | Steep reduction in out of hours contacts for mental health problems at start of lockdown, then recovered but further sustained reduction since April 2021 (Public Health Scotland, n.d).  40% reduction in mental health prescribing, remains below previous levels.  63% reduction in drug and alcohol treatment referrals from March 2020, still 21% lower than historical average (Public Health Scotland, n.d).  Higher levels of anxiety, panic and hopelessness in people with pre-existing mental health problems (Mental Health Foundation, 2020; Mental Health Foundation, 2020a). | Numbers increased of those reporting mental health issues with a few stating it was better. Inequalities widened as those who were identified as vulnerable became more so  Services reduced or moved online. Impact on population groups such as children and young people (Mind Cymru, 2021; Senedd Research, 2020). |
| Those with disabilities including learning challenges | - Impact of disrupted support services | - Those who usually rely on others to provide care and support in the home may have faced difficulties in obtaining this due to movement restrictions. - Opportunities to create diversity in the workforce and enable people with disabilities to join the workforce | Reported reduction in care provision during the pandemic, difficulties with consistency of care, pressure on unpaid carers (The Scottish Parliament, 2020; Disability Equality Scotland, 2020). | Increased negative impact on mental health and wellbeing; access to health and care services and essential services such as shopping (Welsh Government, 2022; Ebuenyi et al, 2020). |
| Homeless people | - Maybe unable to self-isolate, impact of disrupted support services | - Protecting transmission in homeless people by housing them could lead to an end in street sleeping | The number of homelessness applications declined but number of people in temporary accommodation increased. Applications due to being asked to leave or a household dispute increased but those due to loss of accommodation reduced (as evictions were restricted) (Scottish Government, 2021a). | There was an end to street sleeping as homeless populations were housed (Woodfine et al, 2021). |
| People in criminal justice system | - Difficulty of isolation in prison setting, loss of contact with family | - - | Reports of COVID-19 outbreaks in prisons (HMIPS, 2021). |  |
| Undocumented migrants | - May have no access or be reluctant to engage with health services | - - | SG made funding available to organisations to support people with NRPF (Scottish Government, 2020b). No data available on use of this. |  |
| Workers | - Workers on precarious contracts or self-employed – significant risk of adverse impacts from loss of work and no income | - Those who work in sectors which have closed due to restrictions, resulting in people losing jobs or experiencing reduced income. - Key workers who have continued working have been placed at increased risk of contracting the virus and of experiencing mental health impacts such as anxiety and distress. | Impact on economy and employment noted below.  Higher risk of severe Covid-19 among essential workers – healthcare X7, social care and transport X2 increase (Mutambudzi et al, 2021).  High levels of mental distress and suicidality among people who are unemployed or in insecure employment (Mental Health Foundation, 2020). | Reports of health professionals leaving the workforce (The Conversations, 2021).  Stress and burnout in some health and care professionals (Robling and Cannings-John, 2021).  Numbers of people furloughed and becoming unemployed (Welsh Government, 2021a). |
| People with low income | - Impacts will be particularly severe for people on low incomes, who already have poorer health and are more likely to be in insecure work without financial reserves | - Low-income households have been disproportionately affected in a number of ways, including economic and financial impacts; some impacts have been partially mitigated by the national financial support measures such as furloughing. | People on low income have borne disproportionate direct and indirect impacts including higher age standardised death rates from Covid-19, more mental distress (Mental Health Foundation, 2020), increases in educational inequality (data below), financial distress and hardship. Higher income households are more likely to have saved money, increasing economic inequality (Scottish Government, 2020). | Increased claimant count  Reduced income for many workers, particularly low-income groups (ONS, 2021).  Increased levels of household debt in some particular those who were already vulnerable (Welsh Parliament, 2021). |
| People in institutions | - Care homes, special needs facilities, prisons, migrant detention centres, cruise liners – these institutions may act as amplifiers | - Care homes | Reports of covid-19 outbreaks in prisons (HMIPS, 2021).  In 2020 and 2021 29% of COVID-19 deaths occurred in a care home, compared with 21% of non-COVID-19 deaths (National Records of Scotland, 2022). | Increased number of excess deaths due to COVID-19 in care home settings (ONS, 2021b). |

**Supplementary Table S2: Predicted and observed health impacts of the COVID-19 lockdowns on the wider determinants of health**

| **Determinant of health and wellbeing / Pathway of impact** | **Predicted health impacts in Scottish HIA** | **Predicted health impacts in Welsh HIA** | **Observed health impacts in Scotland** | **Observed health impacts in Wales** |
| --- | --- | --- | --- | --- |
| Economic impacts | - Income losses for workers unable to work - Longer term increase in unemployment if businesses fail - Recession | - Negative impacts on the economy resulting in reduced income and spending, increased unemployment, and closure of small businesses; the subsequent health impacts will continue to be felt when measures have been lifted. - Home working has provided greater flexibility for some individuals, enabling individuals to achieve a better work-life balance. | Claimant count (a measure of people on benefits seeking work) increased from 113,100 in March 2020 to 214,900 in May 2020, subsequently partially recovered to 157,100 in October 2021.  GDP fell sharply and in April 2020 was 22.2% below pre-pandemic level in February 2020. It then recovered, by Sep 2021 was just 1.1% below pre-pandemic.  Unemployment rate in Q2 2021 is similar to Q1 2020 but around 3% of employments still supported by Coronavirus Job Retention scheme in Sep 2021  In May 2020 a third of people reported drop in income, 8% difficulty paying bills (Scottish Government, 2020c). | Increased claimant count  Reduced income for many workers, particularly low-income groups (ONS, 2021).  Increased levels of household debt in some particular those who were already vulnerable (Welsh Parliament, 2021).  Increased numbers working from home (NICW, 2020).  Rise in people thinking of leaving workforce (NHS Confederation, 2021).  Increased use of food banks (The Trussell Trust, 2021).  Closure of businesses in key sectors including non-essential retail  Reduced GDP and levels of trade (ONS, 2021c; ONS, 2021d). |
| Social isolation | - Lack of social contact, particularly for people who live alone and have lower access to digital connectivity | - All groups have been affected by reduced interpersonal and social contact, resulting in feelings of isolation and loneliness. - A rapid increase in use of digital technology, which has helped people to stay connected with loved ones, continue working, continue with their education, and access key services (such as healthcare). - The strengthening of family and friendship bonds, for example through increased contact through social media and digital technology. | SG surveys between May and July 2020 found 53-59% adults reported feeling lonely. Between July 2020 and Dec 2021 this was between 44% and 54% (Scottish Government, 2022).  During the lockdown period 32% of people reported changing to working at home (Scottish Government, 2022a), by December 2020 this remained at 31% (Scottish Government, 2020).  Most people reported phoning/video calling/messaging more than pre-pandemic (75% in May 2020, 60% in December 2020) (Scottish Government, 2021b). | High levels of loneliness and distress reported in surveys across all age ranges (WCPP, 2021; Groarke et al, 2020; Public Health Wales, 2020).  Increase in the use of digital technology to connect with family (Public Health Wales, 2020)  Increased connectedness with family and friends and neighbours (Public Health Wales, 2020) |
|  | - Difficulty accessing food and other supplies | - The protection of those who are vulnerable, for example by providing food for children who are eligible for free school meals. | Trussell trust reported 33% increase in foodbanks parcels across UK in 2020/21 compared to previous year, but 7% decrease in Scotland. 8% increase in Wales (Scottish Government, 2021b). Independent food banks in Scotland reported 129% rise in foodbank use Feb to April 2020 (The Trussell Trust, 2021). | Free school meals continued to be made available (Welsh Government, 2021b). |
| Family relationships | - Home confinement may increase family violence and abuse. | - *(potential for abuse noted under populations above)* | 4% increase in police records of domestic abuse incidents in 2020-21 compared with previous year (House of Commons Library, 2021).  Increase in child protection referrals and inter-agency referral discussions and higher proportion of complex cases (Scottish Government, 2021c).  Agencies reported increase in risk, frequency and intensity of abuse in lockdown (Scottish Government, 2021d) and beyond (Scottish Government, 2020c).  In December 2020 53% parents reported feeling stressed about juggling childcare and work, 44% felt too much pressure to support home schooling (Scottish Government, 2021e). | Increase in domestic abuse and child abuse *(see above in women, children and young people)* |
|  | - Potential exploitation of young people not in school | - *(Potential for abuse noted under populations above)* | 6% increase in reports of online child sexual abuse crimes in 2020/21 compared with previous year (Scottish Government, 2020d). |  |
| Health-related behaviours | - Potential for increased substance use, increased online gambling and a rise in unintended pregnancies. - Reduction in physical activity levels as sports facilities closed and less utilitarian walking and cycling | - An increase in health harming behaviours such as snacking and an increase in alcohol consumption. - An increased appreciation of the importance of physical activity, including in promoting mental well-being. For some of the population, physical activity levels have increased. | Overall mixed, with some people adopting healthier behaviours but others less healthy behaviour.  Alcohol – 23% of people self-reported drinking more, and only 14% less (Scottish Government, 2020), but repeated cross sectional studies found increased drinking at home but less on-trade, so overall consumption unchanged (Public Health Scotland, 2021).  Diet – OAS poll in May 2020 found 53% people were boredom eating more, 7% less; 47% eating more confectionary, 13% less; 38% eating more savoury snacks, 10% less. But 43% cooking more from scratch, 4% less and 44% fewer takeaways, 12% more (Obesity Action Scotland, 2020).  Physical activity – OAS poll in May 2020 found 31% people doing less indoor activity, 19% more; 35% doing less outdoor activity, 37% more (Obesity Action Scotland, 2020). English survey found overall 30% reduction in adults reporting any PA, with reduction in utilitarian travel and sports, though increase in gardening and recreational walking. Polarisation with largest decline in younger ages, non-white ethnic groups and unemployed (Obesity Action Scotland, 2020).  Gambling – only 2% of Scots reported gambling more in May 2020, and 6% less (Scottish Government, 2020). UK wide data from Gambling Commission shows overall increase in online bets of 32% in May 2020 compared with March 2019, with fall in real event betting but increase in other bets.  Unintended pregnancies – overall increase of 1.5% in terminations in 2020 compared with 2019 (Public Health Scotland, 2020). | Changes in gambling behaviour - sports cancelled and closure of venues but increased online betting among some (Wardle et al, 2021).  Increased alcohol use in people with alcohol disorder and overall in UK (Kim et al, 2020; Sallie et al, 2020).  Increase in numbers with alcohol related deaths (Kim et al, 2020).  Mixed reports from surveys of physical activity (Public Health Wales, 2020). |
| Disruption to essential services | - Direct impacts on health and social care demand - Unwillingness to attend healthcare settings may impact care of other acute and long-term conditions - Loss of workforce may affect essential services | - Reduced use and / or reduced access to some health and care services, such as hospital Emergency Departments, and the suspension of a number of healthcare interventions, potentially leading to increased morbidity and mortality from non-COVID-19 health conditions. | Weekly planned hospital admissions have remained consistently below 2018-19 average. Peak reduction in April 2020 when admissions were 73% lower than historical average, by Nov 2021 still 12% lower (Scottish Government, 2020).  Compared with 2019, 33% reduction in cancer notifications in April – June 2020, 17% reduction in July – Sep 2020. Suggests delayed/missed diagnoses.  Reduction in coronary angiographies from March 2020.  Reduction in social care support (The Scottish Parliament, 2020). | Reduction in outpatient and elective care. Increased waiting time for elective care (Welsh Parliament, 2021a).  Reduction in elective and emergency non COVID-19 care. Numbers presenting at A and E dramatically reduced but then rapidly increased to higher levels than previously (Oomman and Todd, 2021).  Increased use of digital technology to enable services to be delivered (Budd et al, 2020). |
| Disruption to education | - Loss of education and skills, particularly for young people at critical transitions - Likely increase in educational inequalities from reliance on home schooling |  | Declining overall primary school attainment and increasing attainment gap (Scottish Government, 2021)  Overall attainment of CfE levels for numeracy declined from 79.1% in 2018/19 to 74.7% in 2020/21 (had been increasing pre-pandemic)  Overall attainment of CfE levels for literacy declined from 72.3% in 2018/19 to 66.9% in 2020/21 (had been increasing pre-pandemic)  Attainment gap in numeracy increased from 16.8pp in 20218/19 to 21.4pp in 2020/21 (had been declining pre-pandemic)  Attainment gap in literacy increased from 20.7pp in 20218/19 to 24.7pp in 2020/21 (had been declining pre-pandemic)  SQA attainment hard to compare with previous years due to the change in assessment model. Marked fall in difference between SIMD 1 and 5 in A-C attainment, but not A attainment, for Nat5, Higher and AH in 2020 and 2021 compared with previous years. Eg Higher difference of 21.9pp for A in 2019 and 22.1pp in 2021; for A-C 16.9pp in 2019, 7.9pp in 2021 (Scottish Government, 2021). | Evidence of increased educational inequality and mental wellbeing (Public Health Wales, 2021).  Continued free school meals and access to laptops (Welsh Government, 2021b). |
| Traffic, transport and green and blue infrastructure | - Reduced aviation and motorised traffic with reduced air pollution, noise, injuries, and carbon emissions in short term - Restricted public transport may reduce access for people without a car - Longer term reluctance to use public transport may increase use of private cars - Restricted access to greenspace, which has positive physical and mental health impacts | - Reduced car use and traffic, along with improved air quality and reduced NO2 emissions. - Reduced public transport use, which is likely to continue with the easing of restrictions; this is anticipated to result in an increase in the number of car journeys. This could impact on achieving active and sustainable travel policy goals. | Fall in all journeys, but then rise in car journeys faster than public transport. By June 2021 car journeys similar to pre-pandemic and has remained at that level, by Sep 21 bus and rail journeys are 35% and 50% respectively lower than pre pandemic (Transport Scotland, 2021).  In August 2021 36% of people stated an intention to avoid public transport and use a car more often a year from now.  46% stated an intention to travel less by air (Transport Scotland, 2021a).  In May 2020, 48% of adults had visited greenspace in past 4 weeks, this rose to 63% by Nov 2020, 67% in April 21 (Public Health Scotland, 2021a). | Surveys show concern about use of Public Transport  Some transport services taken back into national government ownership.  Post lockdown increased car use to 90% of pre-pandemic, Public Transport stayed low  Reduced use of greenspace especially older people, low incomes(Welsh Parliament, 2021b). |
| Social disorder | - Potential for unrest if supplies run out or if there is widespread discontent about the response | - A reduction in overall crime rates. - Increased trust in the police | Recorded crime overall in 2020/21 was unchanged from 2019/20, but there were reductions in violent and sexual crimes, dishonesty, fire raising and vandalism. 9% of all crimes were breaches of coronavirus restrictions (Scottish Government, 2021f).  National Community Tensions Team reported (UK wide) heightened tension, protest activity and some hostile confrontations by people opposed to masks and other responses (Police Scotland, 2021). But no significant unrest in Scotland. | Very limited unrest. Few fined for breach of lockdown rules (NPCC, 2021).  Rates of crime fell particularly burglary (ONS, 2020a).  Trust in the police remained high in Wales (Public Health Wales, 2020). |
|  | - Harassment of people believed to be at risk of transmitting the virus |  | 3.9% increase in hate crime in 2020/21 (Police Scotland, 2021).  Many anecdotal reports of harassment affecting people of Asian ancestry and disabled people. See e.g. DES poll of disabled people (Disability Equality Scotland, 2020) |  |
| Psychosocial impacts | - High level of public fear and anxiety | - Social media use has increased the spread of misinformation and feelings of stress and panic. - Poorer mental well-being through fear of the virus; through the stress of working in patient and public facing roles; through increased social isolation; through increased stress of being ‘furloughed’ and working in a sector closed down and reduced income. | Surveys report high levels of anxiety eg SG wellbeing survey in May 2020 29% reported high anxiety level, compared with 20% in ONS benchmark survey. 40% reported feeling more anxious than typical pre-pandemic day, 11% less anxious (Scottish Government, 2020). | High levels of anxiety in surveys (Mind Cymru, 2021).  Many self-reported surveys show social isolation, fear of the virus, fear for children’s and own future and economic impact of the lockdowns and home working are leading to increasingly poor mental wellbeing in the population (Mind Cymru, 2021; Public Health Wales, 2020). |
|  | - Community cohesion could increase as people respond collectively | - Mobilisation of society as a whole to protect those who are more vulnerable, contributing to increased community cohesion and resilience. | In May 2020, 81% reported that if they needed help, they could rely on someone in the neighbourhood (Scottish Government, 2020). Similar in Dec 2020 at 83% (Scottish Government, 2021b).  Many reports of support from voluntary and community sector organisations eg Edinburgh Community Health Forum (Edinburgh Community Health Forum, 2020). | Many reports of community organisations and individuals offering support with increased levels of volunteering – 27% in Wales during lockdowns (Public Health Wales, 2020). |
| Living conditions |  | - Crowded or poor housing quality exacerbating existing health conditions and negatively impacting on mental well-being. - There has been rapid action to place those who are homeless in accommodation. | The number of homelessness applications declined but number of people in temporary accommodation increased. Applications due to being asked to leave or a household dispute increased but those due to loss of accommodation reduced (Scottish Government, 2021a).  In April/May 2020 6% reported more people in the property than pre-pandemic, 3% reported fewer people in the property (Scottish Government, 2020). | Some have been comfortable in lockdown in their homes, but many have not. Financial protection measures have enabled many tenants and homeowners to remain in their homes (Woodfine et al, 2021). |

**References**

Age Cymru. (2020). Experiences of people aged 50 or over in Wales during the first COVID-19 lockdown, and the road to recovery. Available at: <https://www.ageuk.org.uk/globalassets/age-cymru/documents/covid-19-survey/experiences-of-people-aged-50-or-over-in-wales-during-the-first-covid-19-lockdown-and-the-road-to-recovery---october-2020-eng.pdf> (Accessed: 24 March, 2022).

BBC. (2021). Covid in Scotland: People are treating us like the disease. Available at: <https://www.bbc.co.uk/news/uk-scotland-edinburgh-east-fife-56113045> (Accessed: 16 March 2022).

BBC. (2021a). Deaths of people treated under the Mental Health Act rose during pandemic. Available at: <https://www.bbc.co.uk/news/uk-politics-59336579> (Accessed: 24 March, 2022).

BMJ. (2021). ‘Covid-19: Children born during the pandemic score lower on cognitive tests, study finds’. *BMJ* 274:n2031

Budd, J., Miller, B.S., Manning, E.M. *et al.* (2020). ‘Digital technologies in the public-health response to COVID-19’. *Nat Med* **26,**1183–1192. <https://doi-org.mu.idm.oclc.org/10.1038/s41591-020-1011-4>

Cresswell, K., Barton, E., Snowdon, L., Newbury, A., and Cowley, L. (2021). A Health Needs Assessment: The impact of COVID-19 on children and young people’s experiences of violence and adverse childhood experiences. Available at: <https://phwwhocc.co.uk/wp-content/uploads/2021/07/Executive-Summary_The-impact-of-COVID-19-on-children-and-young-peoples-experiences-of-violence-and-adverse-childhood-experiences.pdf> (Accessed: 24 March, 2022).

Davenport, M.H., Meyer, S., Meah, V.L., Strynadka, M.C. and Khurana, R. (2020). ‘Moms are not OK: COVID-19 and Maternal Mental Health’. *Front. Glob. Womens Health* <https://doi.org/10.3389/fgwh.2020.00001>

Disability Equality Scotland. (2020). Your Say on Disability. Available at: <https://yoursayondisability.scot/weekly-poll-social-care-week-beginning-17-august-2020/> (Accessed: 16 March 2022).

Ebuenyi ID, Smith EM, Holloway C*, et al*. (2020). ‘COVID-19 as social disability: the opportunity of social empathy for empowerment’. *BMJ Global Health***5:**e003039

Edinburgh Community Health Forum. (2020). The Contribution of Edinburgh Community Health Forum Member Organisations to the COVID-19 Response. Available at: <http://www.echf.org.uk/wp-content/uploads/2020/11/The-contribution-of-ECHF-to-the-COVID-19-response.pdf> (Accessed: 16 March 2022).

Groarke, J. M., Berry, E., Graham-Wisener, L., McKenna-Plumley, P. E., McGlinchey, E., & Armour, C. (2020). ‘Loneliness in the UK during the COVID-19 pandemic: Cross-sectional results from the COVID-19 Psychological Wellbeing Study’. *PloS one*, *15*(9), e0239698. https://doi.org/10.1371/journal.pone.0239698

Hajek, A., König, H.-H. (2021). ‘Social Isolation and Loneliness of Older Adults in Times of the COVID-19 Pandemic: Can Use of Online Social Media Sites and Video Chats Assist in Mitigating Social Isolation and Loneliness?’ *Gerontology* 67:121-124. doi: 10.1159/000512793

HMIPS. (2021) HM Chief Inspector’s Annual Report 2020-21. Available at: <https://www.prisonsinspectoratescotland.gov.uk/sites/default/files/publication_files/HM%20Chief%20Inspectors%20Annual%20Report%202021-22%20r.pdf> (Accessed: 16 March 2022).

House of Commons Library. (2021). Food banks in the UK. Available at: <https://researchbriefings.files.parliament.uk/documents/CBP-8585/CBP-8585.pdf> (Accessed: 16 March 2022).

Institute for Fiscal Studies. (2020). Parents, especially mothers, paying heavy price for lockdown. Available at: <https://ifs.org.uk/publications/14861> (Accessed: 24 March, 2022).

Kim, J.U., Majid, A., Judge, R., Crook, P., Nathwani, R., Selvapatt, N., *et al*. (2020). ‘Effect of COVID-19 lockdown on alcohol consumption in patients with pre-existing alcohol use disorder’. *The Lancet Gastroenterology and Hepatology* 5:10, 886-887.

Mental Health Foundation. (2020). Coronavirus: The divergence of mental health experiences during the pandemic. Available at: <https://www.mentalhealth.org.uk/sites/default/files/MHF%20The%20COVID-19%20Pandemic%202.pdf> (Accessed: 16 March 2022).

Mental Health Foundation. (2020a). Mental health impacts of the Covid-19 pandemic in Scotland on vulnerable groups. Available at: <https://www.mentalhealth.org.uk/sites/default/files/MHF-Impact-Covid-19-Pandemic-Scot.pdf> (Accessed: 16 March 2022).

Mind Cymru. (2021). Coronavirus: the consequences for mental health in Wales. Available at: <https://www.mind.org.uk/media/8961/the-consequences-of-coronavirus-for-mental-health-in-wales-final-report.pdf> (Accessed: 24 March, 2022).

Mutambudzi, M., Niedzwiedz, C., Macdonald, EB, et al. (2021). ‘Occupation and risk of severe COVID-19: prospective cohort study of 120 075 UK Biobank participantsOccupational and Environmental Medicine’. 78:307-314. <https://oem.bmj.com/content/78/5/307>

National Infrastructure Commission Wales. (2020). Digital Communications Infrastructure in Wales. Available at: <https://gov.wales/sites/default/files/publications/2020-12/digital-communications-infrastructure-in-wales-report-and-recommendations.pdf> (Accessed: 24 March 2022).

National Records of Scotland. (2022). Deaths involving coronavirus (COVID-19) in Scotland. Available at: <https://www.nrscotland.gov.uk/covid19stats> (Accessed: 16 March 2022).

NHS Confederation. (2021). Real risk that thousands of NHS staff will leave unless they are allowed to recover. Available at: <https://www.nhsconfed.org/news/real-risk-thousands-nhs-staff-will-leave-unless-they-are-allowed-recover> (Accessed: 24 March, 2022).

NPCC. (2021). Update on Coronavirus FPNs issued by police. Available at: <https://news.npcc.police.uk/releases/update-on-coronavirus-fpns-issued-by-police> (Accessed: 24 March, 2022).

Obesity Action Scotland. (2020). Lifestyle of Scotland’s People since the Coronavirus Outbreak: Summary report. Available at: <https://www.obesityactionscotland.org/media/1467/polling-summary-report-2805.pdf> (Accessed: 16 March 2022).

Office for National Statistics. (2021). Labour market overview, UK: February 2021. Available at: <https://www.ons.gov.uk/employmentandlabourmarket/peopleinwork/employmentandemployeetypes/bulletins/uklabourmarket/february2021#:~:text=In%20January%202021%2C%20726%2C000%20fewer,lower%20than%20the%20previous%20quarter> (Accessed: 24 March, 2022).

Office for National Statistics. (2021a). Coronavirus (COVID-19) and the different effects on men and women in the UK, March 2020 to February 2021. Available at: <https://www.ons.gov.uk/peoplepopulationandcommunity/healthandsocialcare/conditionsanddiseases/articles/coronaviruscovid19andthedifferenteffectsonmenandwomenintheukmarch2020tofebruary2021/2021-03-10> (Accessed: 24 March, 2022).

Office for National Statistics. (2021b). Deaths involving COVID-19 in the care sector, England and Wales: deaths registered between week ending 20 March 2020 and week ending 2 April 2021. (Available at: <https://www.ons.gov.uk/peoplepopulationandcommunity/birthsdeathsandmarriages/deaths/articles/deathsinvolvingcovid19inthecaresectorenglandandwales/deathsregisteredbetweenweekending20march2020andweekending2april2021#leading-cause-of-death-among-care-home-residents> (Accessed: 24 March, 2022).

Office for National Statistics. (2021c). GDP first quarterly estimate, UK: October to December 2020. Available at: <https://www.ons.gov.uk/economy/grossdomesticproductgdp/bulletins/gdpfirstquarterlyestimateuk/octobertodecember2020#:~:text=1.-,Main%20points,16.1%25%20growth%20in%20Quarter%203.&text=Over%20the%20year%202020%20as,in%20UK%20GDP%20on%20record> (Accessed: 24 March, 2022).

Office for National Statistics. (2021d). UK trade: January 2021. Available at: <https://www.ons.gov.uk/economy/nationalaccounts/balanceofpayments/bulletins/uktrade/january2021> (Accessed: 24 March, 2022).

Office for National Statistics. (2020). Domestic abuse during the coronavirus (COVID-19) pandemic, England and Wales: November 2020. Available at: <https://www.ons.gov.uk/peoplepopulationandcommunity/crimeandjustice/articles/domesticabuseduringthecoronaviruscovid19pandemicenglandandwales/november2020> (Accessed: 24 March, 2022).

Office for National Statistics. (2020a). Coronavirus and crime in England and Wales: August 2020. Available at: <https://www.ons.gov.uk/peoplepopulationandcommunity/crimeandjustice/bulletins/coronavirusandcrimeinenglandandwales/august2020> (Accessed: 24 March, 2022).

Oomman, S., Todd, E. (2021). ‘Impact of COVID-19 lockdown on A&E performances in an NHS Foundation Trust’. *Postgraduate Medical Journal***97:**48-50.

Public Health Scotland. (2021). Changes in alcohol consumption in Scotland during the early stages of the COVID-19 pandemic: Descriptive analysis of repeat cross-sectional survey data. Available at: <https://www.publichealthscotland.scot/media/2983/changes-in-alcohol-consumption-in-scotland-during-the-early-stages-of-the-covid-19-pandemic.pdf> (Accessed: 16 March 2022).

Public Health Scotland. (2021a). COVID-10 Green and Open Space Use in Spring 2021 (Wave 3). Available at: <https://www.gla.ac.uk/media/Media_805950_smxx.pdf> (Accessed: 16 March 2022).

Public Health Scotland. (2020). Termination of pregnancy statistics. Available at: <https://publichealthscotland.scot/publications/termination-of-pregnancy-statistics/termination-of-pregnancy-statistics-year-ending-december-2020/> [https://doi.org/10.1016/j.lanepe.2021.100265](https://doi-org.mu.idm.oclc.org/10.1016/j.lanepe.2021.100265)

Public Health Scotland. (n.d). COVID-19 wider impacts on the health care system. Available at: <https://scotland.shinyapps.io/phs-covid-wider-impact/> (Accessed: 16 March 2022).

Police Scotland. (2021). Quarter 4 Performance. Available at: <https://www.scotland.police.uk/spa-media/zrmixxpv/q4-performance-report-final-quarter-4.pdf> (Accessed: 16 March 2022).

Public Health Wales. (2021). Children and young people’s mental well-being during the COVID-19 pandemic. Available at: <https://phw.nhs.wales/publications/publications1/children-and-young-peoples-mental-well-being-during-the-covid-19-pandemic-report/> (Accessed: 24 March, 2022).

Public Health Wales. (2020). How are we doing in Wales? Public Engagement Survey on Health and Wellbeing during Coronavirus Measures. Available at: <https://phw.nhs.wales/topics/latest-information-on-novel-coronavirus-covid-19/how-are-you-doing/week-4-report-how-are-we-doing-in-wales/> (Accessed: 24 March, 2022).

Refuge. (2021). A year of lockdown: Refuge releases new figures showing dramatic increase in activity. Available at: <https://www.refuge.org.uk/a-year-of-lockdown/> (Accessed: 24 March, 2022).

Robling, M., and Cannings-John, R. (2021). What are the risks for Domiciliary Care Workers in Wales from COVID-19? Available at: <https://mfr.de-1.osf.io/render?url=https://osf.io/wvzj5/?direct%26mode=render%26action=download%26mode=render> (Accessed: 24 March, 2022).

Sallie, S.N., Ritou, V., Bowden-Jones, H.*, et al*. (2020). Assessing international alcohol consumption patterns during isolation from the COVID-19 pandemic using an online survey: highlighting negative emotionality mechanisms. *BMJ Open*;**10:**e044276. doi: 10.1136/bmjopen-2020-044276

Scottish Government. (2022). Coronavirus in Scotland: Loneliness. Available at: <https://data.gov.scot/coronavirus-covid-19/detail.html#loneliness> (Accessed: 16 March 2022).

Scottish Government. (2021). Achievement of Curriculum for Excellence (CfE) Levels 2020-21. Available at: <https://www.gov.scot/publications/achievement-curriculum-excellence-cfe-levels-2020-21/> (Accessed: 16 March 2022).

Scottish Government. (2021a). Homelessness in Scotland: 2020 to 2021. Available at: <https://www.gov.scot/publications/homelessness-scotland-2020-2021/> (Accessed: 16 March 2022).

Scottish Government. (2021b) Coronavirus (COVDI-19) – impact on well-being: survey summary. Available at: <https://www.gov.scot/publications/impact-covid-19-wellbeing-scotland-work-finances-neighbourhood-support-personal-wellbeing-behaviour-changes/pages/1/> (Accessed: 16 March 2022).

Scottish Government. (2021c). Domestic abuse: statistics recorded by the Police in Scotland – 2020/21. Available at: <https://www.gov.scot/publications/domestic-abuse-recorded-police-scotland-2020-21/pages/4/> (Accessed: 16 March 2022).

[Scottish Government. (2021d). The impact of COVID-19 on children and families in Scotland: Understanding needs and services through local social work data. Available at: https://www.celcis.org/application/files/8516/2763/5115/The_impact_of_COVID19_on_children_and_families_in_Scotland.pdf](file://C:\Users\li120104\AppData\Local\Microsoft\Windows\INetCache\Content.Outlook\TO3KOP7R\Scottish%20Government.%20(2021d).%20The%20impact%20of%20COVID-19%20on%20children%20and%20families%20in%20Scotland:%20Understanding%20needs%20and%20services%20through%20local%20social%20work%20data.%20Available%20at:%20https:\www.celcis.org\application\files\8516\2763\5115\The_impact_of_COVID19_on_children_and_families_in_Scotland.pdf) (Accessed: 16 March 2022).

Scottish Government. (2020). Coronavirus (COVID-19): impact on wellbeing – research. Available at: <https://www.gov.scot/publications/impact-covid-19-wellbeing-scotland/pages/5/> (Accessed: 16 March 2022).

Scottish Government. (2020a). Funding to prevent destitution during Covid-19. Available at: <https://www.gov.scot/news/funding-to-prevent-destitution-during-covid-19/> (Accessed: 16 March 2022).

Scottish Government. (2020b). Coronavirus (COVID-19): domestic abuse and other forms of violence against women and girls – 30/3/20-22/05/20. Available at: <https://www.gov.scot/publications/domestic-abuse-forms-violence-against-women-girls-vawg-during-covid-19-lockdown-period-30-3-20-22-05-20/pages/3/> (Accessed: 16 March 2022).

Scottish Government. (2020c). Coronavirus (COVID-19): domestic abuse and other forms of violence against women and girls during Phase 3 of Scotland’s route map (11 August -11 October. Available at: <https://www.gov.scot/publications/coronavirus-covid-19-domestic-abuse-forms-violence-against-women-girls-during-phase-3-scotlands-route-map-11-august-11-october/pages/3/> (Accessed: 16 March 2022).

Scottish Government. (2021e). Public attitudes to Coronavirus: January update. Available at: <https://www.gov.scot/publications/public-attitudes-coronavirus-january-update/>. (Accessed: 16 March 2022).

Scottish Government. (2021f). Recorded crime in Scotland, 2020-21. <https://www.gov.scot/publications/recorded-crime-scotland-2020-2021/pages/3/> (Accessed: 16 March 2022).

Senedd Research. (2020). Mental health, young people and the pandemic. Available at: <https://research.senedd.wales/research-articles/mental-health-young-people-and-the-pandemic/> (Accessed: 24 March, 2022).

SQA. (2021). Equalities Monitoring Report. Available at: <https://www.sqa.org.uk/sqa/files_ccc/2021-acm-equalities-monitoring-report.pdf> (Accessed: 16 March 2022).

Strain, T., Sharp, SJ, Spiers, A. et al. (2022). ‘Population level physical activity before and during the first national COVID-19 lockdown: A nationally representative repeat cross-sectional study of 5 years of Active Lives data in England’. *The Lancet.* 12. [https://doi.org/10.1016/j.lanepe.2021.100265](https://doi-org.mu.idm.oclc.org/10.1016/j.lanepe.2021.100265)

The Conversation. (2021). Nurses are leaving the profession, and replacing them won’t be easy. Available at: <https://theconversation.com/nurses-are-leaving-the-profession-and-replacing-them-wont-be-easy-166325> (Accessed: 24 March, 2022).

The Scottish Parliament. (2020). How has Covid-19 impacted on care and support at home in Scotland. Available at: <https://archive2021.parliament.scot/S5_HealthandSportCommittee/Inquiries/Care_at_Home_Survey_Results_Nov_2020.pdf> (Accessed: 16 March 2022).

The Trussell Trust. (2021). Trussell Trust data briefing on end-of-year statistics relating to use of food banks: April 2020 – March 2021. Available at: <https://www.trusselltrust.org/wp-content/uploads/sites/2/2021/04/Trusell-Trust-End-of-Year-stats-data-briefing_2020_21.pdf> (Accessed: 16 March 2022).

Transport Scotland. (2021). COVID-19 Transport Trend Data – 20 August – 5 September 2021. Available at: <https://www.transport.gov.scot/publication/covid-19-transport-trend-data-30-august-5-september-2021/> (Accessed: 16 March 2022).

Transport Scotland. (2021a). COVID-19 Public Attitudes Survey Data: Waves 20. Available at: <https://www.transport.gov.scot/publication/covid-19-public-attitudes-survey-data-wave-20/> (Accessed: 16 March 2022).

UK Government. (2021). Hate crime, England and Wales, 2020 to 2021. Available at: <https://www.gov.uk/government/statistics/hate-crime-england-and-wales-2020-to-2021/hate-crime-england-and-wales-2020-to-2021> (Accessed: 24 March, 2022).

Wales Centre for Public Policy. (2021). Loneliness in Wales during the Coronavirus pandemic. Available at: <https://www.wcpp.org.uk/wp-content/uploads/2021/10/Loneliness-in-Wales-during-the-Coronavirus-pandemic.pdf> (Accessed: 24 March, 2022).

Wardle, H. et al. (2021) The impact of the initial Covid-19 lockdown upon regular sports bettors in Britain: findings from a cross-sectional online study. [*Addictive Behaviors*](http://eprints.gla.ac.uk/view/journal_volume/Addictive_Behaviors.html), 118, 106876. (doi: [10.1016/j.addbeh.2021.106876](http://dx.doi.org/10.1016/j.addbeh.2021.106876))

Watson, M., Sarica, S., Parkinson, J., Mitchell, R., Wason, D. (2020). COVID-19 Early Years Resilience and Impact Survey (CEYRIS). Report 1 – Key behaviours in children in Scotland aged 2–7 years during COVID-19. Available at: <https://publichealthscotland.scot/media/2878/report-1-_key-behaviours_ceyris.pdf> (Accessed: 16 March 2022).

Welsh Government. (2022). Coronavirus (COVID-19) and the impact on disabled people. Available at: <https://gov.wales/coronavirus-covid-19-and-impact-disabled-people> (Accessed: 24 March, 2022).

Welsh Government. (2021). Key economic statistics: February 2021. Available at: <https://gov.wales/key-economic-statistics-february-2021> (Accessed: 24 March, 2022).

Welsh Government. (2021a). Coronavirus Job Retention Scheme statistics: 9 September 2021. Available at: <https://gov.wales/coronavirus-job-retention-scheme-statistics-9-september-2021> (Accessed: 24 March, 2022).

Welsh Government. (2021b). Written Statement: Extending Free School Meal entitlement to all primary school children. Available at: <https://gov.wales/written-statement-extending-free-school-meal-entitlement-all-primary-school-children> (Accessed: 24 March, 2022).

Welsh Government. (2020). Complex and long-standing disadvantages exposed by the coronavirus pandemic – report finds. Available at: <https://media.service.gov.wales/news/complex-and-long-standing-disadvantages-exposed-by-coronavirus-pandemic-report-finds> (Accessed: 24 March, 2022).

Welsh Parliament. (2021). Debt and the pandemic. Available at: <https://senedd.wales/media/qfalt0jy/cr-ld14666-e.pdf> (Accessed: 24 March, 2022).

Welsh Parliament. (2021a). Impact of the waiting times backlog on people in Wales who are waiting for diagnosis or treatment. Available at: <https://business.senedd.wales/mgIssueHistoryHome.aspx?IId=38257#:~:text=The%20waiting%20lists%20for%20diagnostic%20and%20therapy%20appointments,start%20treatment%20have%20been%20waiting%20over%209%20months>. (Accessed: 24 March, 2022).

Welsh Parliament. (2021b). Putting the ‘public’ back into public transport. Available at: <https://research.senedd.wales/research-articles/putting-the-public-back-into-public-transport/> (Accessed: 24 March, 2022).

Widnall, E., Winstone, L., Mars, B., Haworth, C., and Kidger, J. (2020). Young People’s Mental Health during the COVID-19 Pandemic. Available at: <https://sphr.nihr.ac.uk/wp-content/uploads/2020/08/Young-Peoples-Mental-Health-during-the-COVID-19-Pandemic-Report.pdf> (Accessed: 24 March, 2022).

Woodfine, L., Green, L., Evans, L., Parry-Williams, L., Heathcote-Elliott, C., Grey, C., Azam, S., and Bellis, M.A (2021). No place like home? Exploring the health and well-being impact of COVID-19 on housing and housing insecurity. Available at: <https://phw.nhs.wales/publications/publications1/no-place-like-home-summary-report/> (Accessed: 24 March, 2022).

Wu, B. (2020) ‘Social isolation and loneliness among older adults in the context of COVID-19: a global challenge’. *glob health res policy* **5,**27. <https://doi.org/10.1186/s41256-020-00154-3>

Youth Link Scotland. (2020). Lockdown Lowdown – what young people in Scotland are thinking about COVID-19. Available at: <https://www.youthlinkscotland.org/media/4534/lockdownlowdown-results-by-demographic-breakdown.pdf> (Accessed: 16 March 2022).
